# Microdamage as a Bone Quality Component: Practical Guidelines for the Two‐Dimensional Analysis of Linear Microcracks in Human Cortical Bone

**DOI:** 10.1002/jbm4.10203

**Published:** 2019-07-17

**Authors:** Victoria M Dominguez, Amanda M Agnew

**Affiliations:** ^1^ Skeletal Biology Research Laboratory The Ohio State University Columbus OH USA; ^2^ Department of Anthropology Lehman College CUNY New York NY USA

**Keywords:** AGING, BONE HISTOMORPHOMETRY, FRACTURE RISK ASSESSMENT, INJURY

## Abstract

Microdamage is a component of bone quality believed to play an integral role in bone health. However, comparability between existing studies is fraught with issues due to highly variable methods of sample preparation and poorly defined quantification criteria. To address these issues, this article has two aims. First, detailed methods for preparation and analysis of linear microcracks in human ribs, specifically addressing troubleshooting issues cited in previous studies, are laid out. Second, new, partially validated criteria are proposed in an effort to reduce subjective differences in microcrack counts and measures, ensuring more comparable results between studies. Revised definitions based on current literature in conjunction with a digital atlas to reduce observer inaccuracy and bias are presented. The goal is to provide a practical methodology for bone biologists and biomechanists to collect and analyze linear microcracks for basic science research. © 2019 The Authors. *JBMR Plus* published by Wiley Periodicals, Inc. on behalf of American Society for Bone and Mineral Research.

## Introduction

Bone health is a question of growing concern as our population continues to age. Though a complicated subject, at its most basic bone health refers to bone's ability to maintain function. The most obvious challenge to skeletal health is bone fracture, which undermines the skeleton's ability to carry out all its required functions. Ubiquitous in all human populations, fracture contributes globally to morbidity and mortality rates; however, increased prevalence and severity in certain demographic groups, such as the elderly and particularly elderly females, suggest that unknown risk factors in these populations contribute to compromised bone health.^1^

Traditional diagnostic tools for assessing bone health and fracture risk rely on bone mineral density (BMD), which essentially measures bone mass, but an increasing body of evidence suggests that BMD provides at best an incomplete picture of bone health.^2^, ^3^, ^4^ Clinical evidence clearly demonstrates that decreased BMD is consistent with increased fracture risk, but there is no indication that increased BMD results in a corresponding decrease in fracture risk.^5^ Furthermore, research indicates that bisphosphonates, the most common drug treatment for osteoporosis, halt bone loss but do not necessarily reduce fracture risk in the long term.^6^, ^7^ Recognizing bone mass alone as insufficient to make accurate fracture risk assessments, many clinicians have adopted tools such as Fracture Risk Assessment Tool (FRAX), designed to predict the 10‐year probability of fracture on the basis of multiple clinical risk factors, in addition to BMD.^8^ Among these risk factors are demographic data such as age, sex, weight, and height, as well as patient history including previous fractures or secondary osteoporosis as a consequence of another disorder (ie, diabetes mellitus, hyperparathyroidism, etc.). Although this approach improves predictive outcomes,^8^ it does so without a thorough understanding of the basic biology driving this variation.

As the paradigm shifts away from a strictly bone quantity based measure in clinical assessment, bone quality has risen as a more nuanced contender for defining bone health, both in the realm of clinicians and researchers.^9^ Bone quality considers bone's mechanical properties, both structural and material, when trying to define health and fracture risk.^10^ Among the various components of bone quality, microdamage holds great potential as a major contributor to variable fracture risk and compromised bone health. Long observed in nonbiological, engineering materials as the result of fatigue, microdamage, usually in the form of linear microcracks, signals the imminent failure of these materials.^11^ Frost^12^ was the first to conclusively identify evidence of microdamage in vivo in bone. He also proposed that these linear microcracks repair via targeted remodeling to maintain skeletal integrity, thus explaining why bone does not regularly fail. Since then, numerous studies have provided support for his hypothesis,^13^, ^14^, ^15^ while simultaneously raising more questions about how microdamage influences other mechanical properties and its role in overall bone health. Thus, much remains to be examined to determine the relative importance of microdamage amongst the litany of existing bone quality components.

### What is the basis of bone quality?

Bone quality encompasses skeletal integrity on multiple levels. Though many and often poorly defined, common consensus indicates that among the most important components of bone quality are trabecular and cortical microarchitecture, microdamage, collagen characteristics, osteocytic network viability, and mineralization,^10^, ^16^ elements that range from the nanoscale to the macroscale of bone. More methodical approaches to studying bone quality variables can help researchers and clinicians improve bone health and fragility assessments, and potentially contribute to the development of targeted treatments.

Bone is a composite material with multiscale, hierarchical organization.^17^ Like any material, bone's strength derives from its structural organization and the material properties of its constituent components. Each level of the hierarchy has its own mechanical properties that form the basis of quality assessments, which in turn contribute to the global properties of the whole bone. The interplay between mass, geometry, and material properties at all scales determines bone's strength and its ability to resist loads applied to it. As a biological material, bone is adaptive, adjusting to meet shifting mechanical demands placed upon the body,^18^ as well as restorative, able to repair itself when damaged. This malleability makes bone structure complex, but highly effective in achieving its functions.

Locomotion demands that bones be both strong and flexible, while remaining light enough to be energetically efficient,^10^, ^19^, ^20^ requirements mechanistically at odds. The solution is bones with a hollow cylindrical structure, ensuring greater stiffness, but with far less mass,^21^ as well as a combination of organic and inorganic components arranged to provide a balance between ductility and stiffness.^22^ As a living tissue, bone alters its structure to meet the demands of activity as they shift throughout the lifespan.^18^ Functional adaptation does not create ideal skeletal morphology, but rather a morphology that is likely “good enough” to survive in a given environmental condition. Additionally, material changes to bone affected by altered physiology as the result of genetic, metabolic, and/or environmental factors occur throughout life and interact with structural variation.^23^ These structural and material properties and their functions, interactions, and the changes they undergo are the basis of bone quality.

### What is bone microdamage and why does it occur?

Previous research suggests that^24^, ^25^ microdamage holds great potential for explaining differences in bone strength and fragility among bone quality components. Damage formed at the microscopic level of bone (ie, microdamage) occurs in response to skeletal loading^26^, ^27^ and classifies broadly into three types: microfractures, diffuse damage, and linear microcracks. Microfractures typically occur in trabecular bone,^26^ whereas diffuse damage and linear microcracks are most prevalent in the cortex.^28^ Because the goal of this review is to establish practical guidelines for assessing and analyzing linear microcracks, we concentrate only on that damage type here.

Linear microcracks typically occur in compression, appearing as ellipsoidal shaped planes of separation particularly in interstitial, extra‐osteonal areas of high mineralization within the cortex.^26^, ^28^ Rising in both incidence and length with increasing age, previous research attributes these changes to increased collagen cross‐linking, greater bone mineralization, and increased intracortical porosity and remodeling events,^17^, ^24^, ^29^, ^30^, ^31^ indicating that altered material properties influence a bone's structural response. Inherent repair mechanisms that target these linear microcracks (ie, targeted remodeling) are essential to maintaining skeletal integrity,^15^, ^32^ principally by keeping microcrack propagation in check.^33^ As linear microcracks expand, they sever the connections of the extensive osteocytic canalicular network by shearing the dendritic extensions of viable osteocytes, initiating osteocyte apoptosis and activating remodeling.^34^, ^35^, ^36^ As our physiological systems falter with advancing age, elevated fragility may arise as a consequence of damage accrual outpacing repair.

Microdamage is best understood in terms of fatigue, whereby cyclic loading results in damage accumulation that leads to failure at stresses below normal load thresholds. An example would be dancers who experience lower limb stress fractures due to excessive use.^37^ However, non‐excessive loads such as breathing, which falls within the adapted state of the rib's mechanostat, may also fatigue bone and result in the formation of microdamage.^38^ This raises the question: if damage formation can weaken a bone's overall strength, why does it occur?

At its most basic level, the initiation and propagation of microdamage increases bone toughness, dissipating the energy of applied forces that could otherwise result in catastrophic failure.^26^, ^39^ In addition, some microdamage is essential to bone renewal, as repair mechanisms targeting damaged areas eliminate the compromised areas via their removal and replacement.^13^, ^14^, ^15^ Thus microdamage serves an adaptive role in the skeleton. The ability to initiate microdamage helps bone balance the conflicting demands of stiffness and ductility, whereas targeted repair contributes to maintaining the integrity required of the skeleton in long‐lived, highly mobile species such as humans.^21^

### Why study linear microcracks?

Considering the different varieties of microdamage, why concentrate on linear microcracks here? First and foremost because previous research has indicated that linear microcracks are the most detrimental to bone health and integrity.^24^, ^25^ Diffuse damage is characteristic of younger tissue, as well as chronologically younger individuals. Meanwhile, linear microcrack prevalence is greater in elderly populations, making linear microcracks the more obvious candidate for damage contribution to skeletal fragility.

On a practical level, linear microcracks are the easiest type of cortical microdamage to assess, as they are principally examined via two‐dimensional (2D) methods requiring at minimum staining capabilities and access to light microscopy. Current standards for diffuse damage analysis rely on methods such as scanning electron microscopy because of the submicron scale at which it occurs.^40^ Additionally, there are currently no methods for evaluating cortical microdamage of any kind in vivo, but developing methods will likely detect the larger linear microcracks prior to diffuse damage due to similar issues of resolution.

Last, a number of studies currently exploring aspects of microdamage accumulation already exist, but although some researchers define their criteria for counting and measuring, none have validated these criteria to our knowledge. Validated criteria are essential for reliably comparing the results of studies conducted by different researchers in different settings. Our aim is to build upon the work of previous researchers while standardizing methods that can be of use to the field.

In order to build reliably comparable data for the analysis of linear microcracks, the aims of this review are twofold. First, we lay out in detail methods for preparation and analysis of linear microcracks in human ribs, specifically addressing troubleshooting issues cited in previous studies. Building on existing methods, the Skeletal Biology Research Laboratory (SBRL) at The Ohio State University has honed these techniques during the preparation of several hundred human ribs for analysis. Here, the successful, as well as failed, methods are documented in hopes of furthering the discipline. Second, few existing studies expressly define how and what they include in their microcrack counts, and among those that do, we are unaware of any that have validated their criteria. We propose new, partially validated criteria in an effort to reduce subjective differences in microcrack counts and measures, ensuring more comparable results between studies. Working from existing criteria, we present revised definitions in conjunction with a digital atlas to reduce observer inaccuracy and bias. From this review of the existing literature combined with our own contributions, our goal is to provide a practical methodology for bone biologists and biomechanists to analyze linear microcracks.

## Preparing Samples for Analysis

Preparing undecalcified bone sections for microdamage analysis requires approximately 4 weeks, from initial sampling of the bone to completed slide, as well as a fair amount of equipment and consumables. In this paper, we review the existing methods most often cited in the literature followed by a detailed description of our approach, which is based upon the methods of Burr and Hooser^41^ and honed for the preparation of human rib samples. Since 2012, approximately 1000 slides of human ribs have been prepared in the SBRL for linear microcrack analysis, a process during which various troubleshooting techniques have been developed and tested. Here, current procedures for staining, embedding, sectioning, and mounting human ribs are laid out step by step. Additionally, a complete list of the materials and equipment used in these procedures is provided in the Supporting Information, Part I.

### Staining

The most popular technique for linear microcrack analysis is en bloc staining. First proposed by Frost^12^ and later validated by Burr and Stafford,^42^ en bloc staining distinguishes in vivo damage from artifactual damage due to sample preparation. Basic fuchsin, a fluorescing and ethanol‐soluble dye, is diffused throughout the selected bone samples prior to processing, permeating any open spaces in the bone so that in vivo damage will be stained and can be distinguished from processing damage, which occurs after staining is complete and thus remains unstained. Concerns about dehydration shrinking creating cracks during the staining process were put to rest by Burr and Stafford's^42^ validation, which found that stained ribs had a comparable number of microcracks to unstained controls. In the mid‐1990s, the original protocol was revised, introducing a graded series of alcohols under vacuum to better perfuse stain throughout the bone.^41^ Despite the existence of successful guidelines for staining, all protocols possess issues that require trouble‐shooting with regard to the particularities of a given project and/or laboratory. The SBRL's protocols are based on those outlined by Burr and Hooser^41^ and tailored with the much appreciated aid of Keith Condon at the Indiana University School of Medicine for the preparation of human rib sections, which is the current focus of microdamage analysis at the SBRL. The SBRL protocols are described here, including specific measures used to address problems previously described by Burr and Hooser.^41^

#### Block preparation

Blocks ~2 cm in length are carefully excised from fresh (ie, unembalmed and still fleshed) ribs using a Dremel tool and incised on the pleural and inferior vertebral end surfaces to maintain orientation (the PIV notch). Each section is divested of soft tissue via gentle maceration in a solution of water and detergent in a slow cooker set at low heat for a maximum of 4 hours. Once clean, each rib section is placed in a nylon biopsy bag with a paper label written in pencil (preferably a traditional graphite pencil) and stapled closed. Biopsy bags are permeable, allowing staining to occur in large batches, while maintaining the identification for each section. Pencil is used for specimen labels because once infused with stain ink becomes obscured on the paper labels, whereas once the stained labels dry, pencil remains legible. Prior to staining, samples within their biopsy bags are deposited in a 70% ethanol (EtOH) solution for a minimum of 48 hours. Sometimes staining cannot proceed immediately for any number of reasons (ie, scheduling conflicts, waiting for a larger batch size, etc.), in which case samples may safely remain in the 70% EtOH solution indefinitely until staining can proceed.

#### Preparing the stock solutions

Similar to Burr and Hooser,^41^ basic fuchsin obtained from J.T. Baker (Radnor, PA, USA) is made into 1% stock solutions of 80%, 90%, or 100% EtOH. All stock solutions are made by a gradual introduction of the basic fuchsin granules into the EtOH solutions and then allowed to sit on an active stir plate for a minimum of 24 hours (80% and 90%) or 48 hours (100%) to ensure the dye goes into solution. Each solution should have a volume equivalent to a ratio of at least 10 × staining solution to bone.

#### Staining schedules

Following the 70% EtOH solution, samples are placed directly into a series of graded alcohol solutions under vacuum at 20 inHg (67.7 kPa) over the course of 1 week. Burr and Hooser^41^ highlight that density affects bone's ability to uptake stain, with poorly mineralized bone requiring reduced staining times and highly mineralized bone requiring longer staining periods. This requires differential staining schedules meant to prevent either overstaining or understaining samples, and thus complicating analysis. To address this issue, staining schedules in the SBRL are adjusted based on the chronological age of the samples in a given batch. Although acknowledging that age is not an ideal indicator of bone mineralization, generally speaking, older individuals often have a greater proportion of highly mineralized bone compared to younger individuals. Thus, prior to staining, samples are roughly divided into either a “Young” or “Old” category using an arbitrary cutoff age of 55 years. Having attempted various permutations of age groups and staining schedules, we have found that this is the simplest and most effective approach to minimize understaining or overstaining samples, because it is of course impossible to observe the amount of primary versus secondary bone prior to slide making or to measure degree of mineralization.

Based on the age of the group (“Young” or “Old”), the timing and strength of solutions used varies. The “Old” group requires both more solutions and a longer staining schedule. For the “Old” group, the protocol is as follows:

1. Immerse in 1% basic fuchsin in 80% EtOH for 24 hours.
2. Change solution, immerse in 1% basic fuchsin in 80% EtOH for 24 hours.
3. Change solution, immerse in 1% basic fuchsin in 90% EtOH for 24 hours.
4. Change solution, immerse in 1% basic fuchsin in 100% EtOH for 48 hours.
5. Change solution, immerse in 1% basic fuchsin in 100% EtOH for 48 hours.
6. Thoroughly rinse samples in 100% EtOH to remove excess stain.

For the “Young” group, the protocol is the same but excludes step 2. Though rare in the SBRL, we recommend that if the samples in question are less than 35 years of age, step 6 also be excluded. For all stages in any group, the samples are kept in solution under vacuum at 20 inHg (67.7 kPa). Samples should be periodically stirred to prevent uneven staining. To rinse the samples, they are agitated within their biopsy bags in pure ethanol until minimal dye is released, which is a lengthy process.

### Embedding

In order to maintain the delicate trabecular architecture of the rib during sectioning, samples in the SBRL are embedded in 99% methyl methacrylate (MMA) immediately following staining. MMA is the plastic of choice for many working with undecalcified bone, either stained or unstained, because its hardness is comparable to that of bone, improving the chance of intact sections during and after cutting.^43^ Additionally, embedded samples are easier to store and preserve long‐term than unembedded samples.

#### Infiltration

After thorough dehydration prior to and throughout the staining process, bone samples require an extended period of MMA infiltration prior to embedding.^43^ Once thoroughly rinsed of excess dye, the biopsy bags containing stained samples are immersed in liquid MMA and kept under vacuum for 24 hours. After 24 hours, samples are transferred to a solution of MMA and 4% dibutyl phthalate and kept under vacuum at 20 inHg for a minimum of 5 days, with the vacuum pulled at least every 24 hours. A cold trap vapor condenser should be added to prevent MMA gases from contaminating the pump. Dibutyl phthalate works to moderately soften the MMA and increase its elasticity.^43^ New MMA solutions are not necessary for the infiltration portion of the embedding process and the same MMA is often used to prepare numerous batches before disposal, which usually occurs when reused MMA becomes cloudy with remnant stain from the soaking samples.

#### Preparing the embedding molds

After infiltration, samples and labels are taken from their biopsy bags and the PIV notch in each bone is marked with pencil to make it easily visible through the plastic after embedding. Each sample receives its own mold, previously labeled in pencil on waterproof tape. When selecting embedding molds, make sure the containers are sealable and made of either glass or a plastic that will not react with MMA. Film canisters are recommended because they have tight caps and are cheaply acquired. Molds should be previously prepared with an MMA base of ~5 mm to prevent samples from resting on the bottom of the mold and thus allowing them to be fully suspended in MMA.

#### Embedding procedure

An initiator is required to create the catalyzed MMA to embed the sample. A solution of new MMA and 4% dibutyl phthalate is mixed with 0.25% Perkadox and thoroughly stirred to activate the solution. As MMA cures, it shrinks, so each container must be filled with sufficient catalyzed MMA to more than cover the samples (for our samples, ~10 mL) in order to ensure that the bone remains fully covered once the MMA has set. Any excess activated MMA can be used to create bases in empty molds for future rounds of embedding.

Filled molds are left uncovered under vacuum at 20 inHg (67.7 kPa) for at least 12 hours, then they are sealed and refrigerated overnight. Once cooled, the sealed molds enter a water bath at room temperature, ensuring the water line rests above the contents of the canisters, and the temperature is gradually increased at a rate of 0.3°C every hour until reaching 37°C. At that point, the molds are taken from the water bath and the lids removed to confirm that the MMA has set. If the MMA is still not solid, the molds are resealed and placed back in the water bath until the MMA sets. When the MMA has set, open molds are placed in an oven at 60°C and cured overnight.

### Sectioning blocks

Once cured, embedded sample blocks are cut free and the molds discarded. Tape labels from the discarded molds are transferred to the MMA blocks themselves. Using the PIV notch as a guide, orientation labels are written on the surface of each block (ie, P = pleural, I = inferior, V = vertebral) to maintain orientation during sectioning.

Traditional preparation of undecalcified sections calls for sectioning at a thickness of 250 to 500 µm, then manually grinding samples to 30 to 150 µm (exact measures vary by study).^12^, ^35^, ^42^ Sections are most often cut using a diamond blade wafering saw and ground on a turntable‐style grinder until reaching the desired thickness. Although cutting and grinding may introduce artifactual microdamage, such instances are excluded from counts and analysis by eliminating cracks that are not stained through the depth of the section. However, the use of a diamond wire saw allows sectioning of undecalcified material at a thickness of ~70 to 125 µm and eliminates grinding from the slide‐making process, reducing the opportunity for artifactual damage formation. The SBRL uses a Delaware Diamond Knives, Inc. (Wilmington, DE, USA) diamond wire saw with a 0.22‐mm‐diameter diamond wire to prepare sections for slides. Distilled water is used to keep samples hydrated, preventing the dissolution of the dye and keeping stain contained to the porous locales in the bone.

Undecalcified tissue sections, even at 120 µm, often curl when drying. Thus, once cut, sections are wrapped in a Kimwipe and pressed between two slides with binder clips for at least 48 hours (timing varies depending on the ambient temperature and humidity). This removes excess moisture absorbed during sectioning and ensures that sections dry flat, considerations that ease the slide making process. To maintain orientation of the sections throughout processing, it is recommended that a consistent corner of the embedding material be clipped (ie, inferior/pleural corner) prior to pressing.

### Mounting slides

After pressing, sections are mounted on 1 × 3 inch glass slides with etched labels. The dried, flat sections are trimmed of excess MMA and cleared in xylene prior to mounting. As a mounting medium, the SBRL currently uses Eukitt® (Germany) for its quick curing time, its long‐term preservation, and because it helps maintain stain. Mounting media with long curing times often lead to bubble formation beneath the coverslip, which can compromise slide utility, and long‐term preservation with many media is poor, often yellowing and/or crystallizing so that slides must be remounted within 5 years. Being sure to permeate the bone, three drops of mounting medium are used to coverslip sections. Slides are left to lie flat in dark drying trays for a minimum of 2 weeks. Though slides can be imaged and analyzed immediately, the 2‐week drying period ensures that the mounting medium fully cures, preventing bubble formation beneath the coverslip. When not in use, slides should be stored in a cool, dry, dark place for best preservation.

## Data Collection

Conducting accurate and detailed analyses of linear microcracks in 2D is best done through a combination of high‐resolution still images and live observation on the microscope. This section provides recommendations for imaging basic fuchsin‐stained slides, revised classification criteria, and partial validation results, and considerations for data collection using still images. Though developed on human ribs, the information and techniques described here apply to linear microcrack analyses of any skeletal element.

### Imaging

Many factors influence the success of imaging for data analysis, including slide quality and the camera, microscope, and computing power available. Two considerations for any imaging regardless of these features, magnification and illumination, are presented here.

#### Magnification

Selecting magnification for data collection depends on the data to collect and the quality of the camera available. The smaller the feature, the higher the imaging resolution needed for accuracy.

For microcrack analysis in the SBRL, ribs are analyzed and imaged at ×200 magnification. Previous studies used a variety of magnifications for microdamage analysis, though most fall between ×150 and ×200 (see Table 1). Based on reported average lengths, this appears to be sufficient resolution to capture most visible linear microcracks and allows our data to be more directly comparable to existing studies (see Agnew and colleagues^44^ for a more detailed discussion of how magnification differences may influence linear microcrack quantification).

**Table 1:** Magnifications, Illuminations, Bones, and Sampling Regions for Some Linear Microcrack Analyses From the Literature

Reference	Magnification	Illumination	Bone	Sampling
Frost^12^ (1960)	×50^a^	BF	Human rib	Complete cross‐section
Burr and Stafford^42^ (1990)	×100	BF	Human rib	Complete cross‐section
Schaffler and colleagues^61^ (1995)	×200	BF	Human femur	Principal anatomical axes (anterior, poster, medial, lateral)
Boyce and colleagues^62^ (1998)	×125	BF	Human tibia	Beam cross‐sections representing tensile, neutral axis, and compressive regions
Burr and colleagues^50^ (1998)	×150	BF	Dog femur	Complete cross‐section
Lee and colleagues^45^ (1998)	×125, ×225	BF, FL	Human rib	Complete cross‐section
Huja and colleagues^63^ (1999)	×125, ×250	BF, FL	Dog femur	Complete cross‐section
Danova and colleagues^51^ (2003)	×200	BF	Rat ulna	Complete cross‐section
Diab and Vashishth^57^ (2005)	×125	BF	Bovine tibia	Anterior quadrant
Diab and Vashisth^28^ (2007)	×125	BF	Human tibia	Anterior and posterior cortices
Herman and colleagues^35^ (2010)	×400	BF (?)	Rat ulna	Complete cross‐section
Agnew and colleagues^44^ (2017)	×400	FL	Human rib	Complete cross‐section

BF = bright field; FL = fluorescent lighting.

^a^ Magnification based on figure legends because the magnification for analysis is never expressly stated in the text.

The disadvantage of such high‐resolution photography is the size of generated image files. For example, a complete human rib at ×200 magnification can result in a 2‐Gb tag image file format (TIFF) file. As technology advances, such concerns may become obsolete, but this is not currently the case. Although microcrack analysis can be carried out imaging only regions of damage, such a piecemeal approach is untenable for measuring cross‐sectional geometry variables. Complete cross‐sectional images are preferred, but because these variables are larger, the images can easily be taken at lower magnifications (eg, ×40, ×100), significantly reducing file sizes. Data are comparable across images as long as appropriate scales are included.

#### Illumination

**Figure 1:**
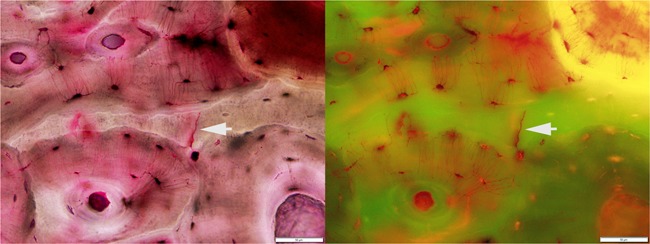
A stained linear microcrack highlighted by a white arrow. The image on the left is under bright field illumination, whereas the image on the right was photographed under FITC fluorescence. The dark pink color in the left image is the basic fuchsin permeating the open spaces, which appears red and in stark contrast to the unstained green portions of the bone in the right image.

Bright field and fluorescent lighting each have advantages and are often used together, both for imaging and live microscopy in the SBRL. Although microcrack analysis can be and is conducted using bright field illumination, Lee and colleagues^45^ showed that the use of FITC fluorescence can improve an observer's ability to see and assess microdamage when using basic fuchsin to stain bone. Pink in bright field illumination, basic fuchsin appears deep orange/red when viewed under fluorescence. Thus regions of the bone infiltrated by basic fuchsin stand out against those that have not absorbed any stain, which appear green under FITC fluorescence (Fig. 1). This can be especially helpful to those new to linear microcrack analysis, because fluorescence emphasizes the contrast between potential microcracks and the surrounding bone more so than bright field illumination. Although identifying potential microcracks is easier with fluorescence, confirming that microcracks open on the surface of a section (see Classification criteria) is easiest in bright field, live on the microscope. For the most accurate results, analysts should combine imaging with live microscopy and actively use both bright field and fluorescent illumination if possible.

### Histomorphometric variables

Histomorphometry is an essential tool for 2D microdamage studies using quantitative methods to analyze bone microarchitecture. Microdamage quantification occurs in different ways from classification by type and location (eg, diffuse versus linear microcracks, lamellar versus interstitial bone, etc.^28^, ^46^) to assessment in relation to features such as porosity or lacunar density,^47^, ^48^ and even measuring the displacement of crack openings.^49^ The most common approach is simple counts and measures of linear microcracks and their areal densities.^30^, ^47^, ^49^, ^50^, ^51^, ^52^, ^53^ Four variables are commonly used in linear microcrack studies: crack number (Cr.N), crack length (Cr.Le), crack density (Cr.Dn), and crack surface density (Cr.S.Dn). These variables and other common histomorphometric measures used in damage analysis are listed and defined in Table 2. Two of these variables, Cr.N and Cr.Le, are direct observations usually taken from images, whereas Cr.Dn and Cr.S.Dn must be calculated using Cr.N and Cr.Le in combination with a measure of bone area.

**Table 2:** Histomorphometric parameters important for linear microcrack analysis in the ribs

Parameter name	Abbreviation	Definition	Unit	Formula^a^
Total area	Tt.Ar	Total area below the periosteum, including the medullary cavity	mm^2^	NA
Endosteal area	Es.Ar	Total area below the endosteum (ie, the medullary cavity)	mm^2^	NA
Cortical area	Ct.Ar	Area of all cortical bone, including pores	mm^2^	Tt.Ar – Es.Ar
Pore area	Po.Ar	The sum of the area of all pores (ie, Haversian canals, resorption spaces)	mm^2^	∑Pore_1_ + Pore_2_ + … + Pore_x_
Bone area	B.Ar	Area of all cortical bone, excluding pores	mm^2^	Ct.Ar – Po.Ar
Crack number	Cr.N	Count of observed linear microcracks	#	NA
Crack length^b^	Cr.Le	A minimum length of measure for each linear microcrack	μm	NA
Crack density^c^	Cr.Dn	Areal density of cracks normalized by a specified bone area	#/mm^2^	Ct.N/B.Ar
Crack surface density^c^	Cr.S.Dn	Surface area occupied by cracks over a specified bone area	μm/mm^2^	Cr.N*meanCr.Le/B.Ar

^a^ The formula used to calculate the derived variables using the observed variables in this table are provided.

^b^ In analysis, mean Cr.Le is generally used, rather than each individual Cr.Le.

^c^ In most literature, areal densities for linear microcracks are calculated using the variable Ct.Ar; however, we propose the use of B.Ar as a more accurate indicator of damage density.

**Figure 2:**
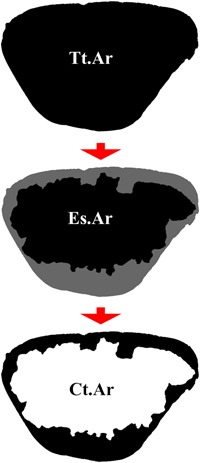
Calculating cortical area. Example of a human rib cross‐section demonstrating how to calculate Ct.Ar. First, the Tt.Ar of the cross‐section is measured, including everything below the periosteal border. Second, the Es.Ar is measured, which is essentially the area of the medullary cavity. Finally, the Es.Ar is subtracted from the Tt.Ar, resulting in the total area of the cortex (Ct.Ar). Ct.Ar = cortical area; Tt.Ar = total area; Es.Ar = endosteal area.

When comparing data across individuals, density variables are particularly useful, because the inclusion of bone area acts as an allometric control. Most literature uses cortical area (Ct.Ar, called “bone area” in some papers, though a distinction is made between those variables here) to calculate areal densities, which is defined as the total cortex examined, including porosity present within the defined boundaries. These boundaries may refer to anything from complete cross‐sections of a bone to a fraction of the cortex. In the ribs, Ct.Ar is calculated by taking the total subperiosteal area of a cross‐section and removing the endosteal area (Fig. 2). Although valuable in many respects, this measure fails to account for intracortical bone loss in the form of porosity, a major concern in the rib especially. Porosity in the cortex consists primarily of bone's vascularizing channels, the Haversian canals, and the resorption spaces that result from osteoclastic activity. In the rib, elevated remodeling and high sensitivity to metabolic changes can result in substantial bone loss within the cortex that Ct.Ar does not measure.^54^ An alternative variable, bone area (B.Ar), calculated by removing porosity area from the cortical area, more accurately reflects the amount of bone present.

Preliminary study in a sample of 10 elderly individuals indicated that Ct.Ar and B.Ar differ significantly, implying that intracortical porosity comprises a significant part of the “area” measured in Ct.Ar.^55^ Considering this, B.Ar's more precise measure of viable cortex may better represent the impact of microdamage on bone fragility than Ct.Ar.^55^ This concern is particularly relevant when viewed in light of evidence showing existing pores as potential stress raisers.^48^ Furthermore, in microdamage studies, accounting for intracortical porosity may significantly influence areal density measures of microcracks. Because calculating bone area requires the time‐consuming collection of porosity measurements, further exploration of the differences between these variables is needed and is currently underway in the SBRL.

### Classification criteria

As stated in the Staining section above, basic fuchsin indiscriminately infiltrates the open spaces within bone, including not only microdamage, but also Haversian systems, Volkmann canals, and canalicular networks. As such, accurately distinguishing microdamage from other features of bone microarchitecture requires familiarity with bone histomorphology. Although some studies have used chelating fluorochromes that selectively bind to microcracks and ease identification,^56^ such methods are still rare and do not resolve some of the quantification issues inherent in microcrack analysis. Specifically, few researchers are explicit in how they determine what to include or exclude as linear microcracks. The earliest guidelines for what should “be accepted as a crack” were laid out by Frost^12^ (Table 3), in an effort to reduce the inherent subjectivity he recognized in microcrack assessment. Since then, however, most papers provide perfunctory descriptions at best of what they count as a microcrack, often citing “linear structures”^51^ or “a sharply defined line”^57^ as the only qualifier for their data collection. The aims of this section are not to impugn the valuable work of prior researchers, but establishing clear criteria for identifying, counting, and measuring linear microcracks can aid in improving the reliability of data collected by observers of various experience levels, regardless of sample preparation, as well as increasing repeatability internally and between researchers.

**Table 3:** Linear Microcrack Criteria as Defined in the Literature

Reference	Criteria
Frost^12^ (1960)	Must be stained through the depth of the section
	Must open visibly onto the surface of the section
	No permeation of stain into the bony substance in the walls of the crack
Burr and Stafford^42^ (1990) [used by Lee and colleagues^45^ (1998);	Intermediate in size, larger than canaliculi but smaller than vascular channels
O’Brien and colleagues^25^ (2005)]	Sharp borders with a halo of basic fuchsin staining around them
	Stained through the depth of the section
	When the depth of focus is changed, the edges of the crack are more deeply stained than the intervening space
Schaffler and colleagues^61^ (1995)	Linear structures with basic fuchsin staining within cracks
Frank and colleagues^30^ (2002); Danova and colleagues^51^ (2003)	Linear structures with basic fuchsin staining around the cracks
Diab and Vashishth^57^ (2005)	Sharply defined line
Herman and colleagues^35^ (2010)	Linear regions of basic fuchsin uptake with sharp boundaries

Thirty years after Frost's^12^ initial guidelines, Burr and Stafford's^42^ validation of the en bloc staining method included an updated set of criteria for counting microcracks (Table 3). The criteria presented in the current study are based in part on the definitions laid out by Frost^12^ and Burr and Stafford,^42^ but include considerations designed to further reduce subjectivity in counting and measuring. In addition to the written descriptions provided here, a photographic atlas composed of representative images illustrating the criteria outlined in Table 4 can be found in the Supporting Information, Part II.

**Table 4:** Linear Microcrack Criteria

A linear microcrack is a sharply defined line with a halo of basic fuchsin. Linear microcracks are more robust in appearance than canaliculi, which often resemble a spider web crisscrossing the bone, but smaller than vascular channels such as Volkmann's canals, which are wider.
Only cracks that visibly open on the surface of the section being analyzed should be counted. When viewed under the microscope, the feature in question should be counted if it appears to open when the depth of focus is changed; if not, it should be excluded. This is best seen in bright field.
Microcracks that are not artifactual will be stained through the depth of the section, appearing darker than the surrounding area.
Single cracks that are distinguishable from tip to tip should be counted as one. Length measurements should follow the path of the crack.
In some cases, cracks appear to coalesce, resulting in what looks like tree branching. In these instances, the longest axis should be counted as one and measured as the primary crack. Branches should be counted as independent cracks, with each branch measured from the point of intersection with the primary crack to their free end.
Additional criteria
Microcracks are sometimes seen to be partially stained. This is attributed to one of two causes: (1) the stain did not fully impregnate the existing microcrack during the staining process, or (2) the existing microcrack continued to propagate during the process of slide preparation after the staining process was completed, resulting in a portion of the microcrack being unstained.
**(1)** If the crack is not stained through the depth of the section, do not count it.
**(2)** If a part of the crack is stained through the depth of the section, the microcrack should be counted, but only the stained portion should be measured.
Debonding of lamellar layers presents with a similar appearance to linear microcracks. This is generally seen between layers of concentric lamellae (within secondary osteons) or longitudinal layers of lamellar apposition (at the periosteal borders of the bone). Debonding should not be counted as microcracks, with two exceptions.
**(1)** If the plane of separation is great enough that it appears open on the surface of the section, it should be counted and measured. This is most reliably determined live on the microscope.
**(2)** If the microcracks run into and propagate through such planes of separation, it should be counted. This happens most often when microcracks break through or deflect around an osteon's reversal line.
Instances where a series of fine lines that are difficult to distinguish are seen to run through each other are known as cross‐hatching. Damage that bears a cross‐hatched appearance is not open to the section surface and cannot be reliably traced, and therefore should not be counted in linear microcrack assessment.

#### Partial validation study

To assess whether the refined criteria improve precision within observers, a sample of 10 human rib cross‐sections prepared for linear microcrack analysis were examined. Slides were prepared and imaged following the protocols laid out above. A single observer, proficient in bone histology, with over 7 years of experience, but a novice in linear microcrack analysis read each slide twice to assess intraobserver error. Readings occurred a minimum of 2 months between observations.

Cr.N and Cr.Le were assessed at ×200 magnification and annotated on previously taken photographs of the ribs under fluorescence. Differences between the observer's first and second reads were assessed by calculating the measurements' coefficient of variation (CV). CV is a standardized measure, usually given as a percentage, of dispersion around the mean calculated as follows: standard deviation/mean * 100. To assess the precision of the revised criteria, the CV of each of the 10 subjects was calculated and rates were deemed acceptable when below 10%.

CVs greater than 10% were found for four subjects in Cr.N and two subjects in Cr.Le (Tables 5 and 6). For Cr.N, in all instances of CVs greater than 10%, the number of cracks counted in the second read was higher than on first observation. The first read of the slides was also the observer's first ever experience reading microcracks. By the time of the second slide reading, two months of exposure to slide reading had transpired, improving the observer's familiarity and ability to identify microcracks. The fact that over one‐half of the slides read were within the acceptable margin indicates that the criteria are useful for internal consistency in a novice analyst. The consistent increase in counts between reads suggests there is a learning curve for assessing linear microcracks, but this must be further explored. The same pattern was not observed in Cr.Le; however, both instances of significant deviation were in slides with low crack densities. Overall, the criteria presented here are promising, but require full‐scale testing, incorporating multiple observers with differing degrees of experience in microcrack analysis to be fully validated.

**Table 5:** Intraobserver CVs for Cr.N

Sample	Read 1	Read 2	CV
1	30	30	0.00
2	2	2	0.00
3	31	66	**36.08**
4	35	46	**13.58**
5	13	14	3.70
6	21	25	8.70
7	5	8	**23.08**
8	101	107	2.88
9	25	35	**16.67**
10	43	43	0.00

CV values in bold are above the 10% threshold for acceptable error.

CV = coefficient of variation; Cr.N = crack number.

**Table 6:** Intraobserver CVs for Cr.Le

Sample	Read 1	Read 2	CV
1	0.0427	0.0402	3.12
2	0.0966	0.0290	**53.80**
3	0.0463	0.0446	1.83
4	0.0485	0.0431	5.91
5	0.0246	0.0451	**29.34**
6	0.0482	0.0519	3.61
7	0.0488	0.0536	4.64
8	0.0510	0.0557	4.46
9	0.0545	0.0466	7.72
10	0.0504	0.0444	6.28

CV values in bold are above the 10% threshold for acceptable error.

CV = coefficient of variation; Cr.Le = crack length.

### Quantification using images

Although use of live microscopy to ensure accurate data collection cannot be emphasized enough, quantification of histomorphometric data often relies on measuring photographic images. Methods for data collection from still images are briefly described in the subsequent sections.

#### Data collection software

Although many software options for collecting data exist, most histomorphometric analysis in the SBRL uses NIH‐funded ImageJ.^58^ ImageJ is freely available online and widely used by a number of laboratories, allowing easy and reliable comparison of data between researchers. Furthermore, creating region of interest (ROI) sets in ImageJ allows users to amass large datasets without duplicating large image files and consuming valuable storage space. Essentially overlay files, ROI sets can store a large number of adjustable selections for a given image at a fraction of the digital space required by permanently annotated images. Last, ImageJ allows the creation and use of customized macros and plug‐ins, which can simplify data collection. Detailed information on the use of ImageJ ROI sets and customized macros for histomorphometric analysis can be found in Dominguez and Agnew.^59^

#### Linear microcrack measures in ImageJ

The SBRL method for collecting linear microcrack data using ImageJ is briefly described here. The slide is assessed on the microscope and all observed microcracks on the entire cross‐section are annotated on the corresponding image file within the software. The segmented or freehand line selection tools are used to trace the path of observed microcracks, with each crack traced and recorded independently. Occasionally, multiple cracks appear within a microscopic field and are not in the same plane of focus on the reference image. In such instances, additional photographs are taken, adjusting the camera's focus so that the visible microcrack is sharply defined and can be accurately measured. Any additional photography is denoted on the reference image. Counts and measures of microcrack data are then combined for each rib prior to data analysis.

## Considerations for Linear Microcrack Work

### When staining en bloc, is it more detrimental to understain or overstain?

**Figure 3:**
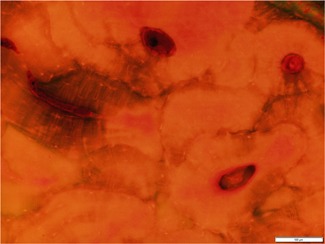
Image of an overstained bone that is imaged under fluorescence. The excess of stain makes it difficult to distinguish fine features in the bone.

Although achieving a perfect stain is desired, in reality there is no guarantee. Overstaining, due either to too high a stain concentration or too long soaking in stain, can make slides difficult to read. As noted, basic fuchsin does not preferentially stain microcracks, so any pores and regions of collagenous matrix can take up high quantities of stain, making distinguishing damage from cortex difficult, especially under fluorescent illumination (Fig. 3). On the other hand, slides that fail to soak in solution for enough time may result in sections which do not absorb any stain, creating artificially low microdamage measures. Thus, it seems preferable to err on the side of overstaining rather than understaining.

### What causes stain to appear patchy and uneven in some sections?

Stain uptake can be irregular due to irregular mineralization in the cortex. When this is the case, there is no solution we are aware of, though it does convey some information about the bone health of the individual in question. More often, this patchy appearance likely results from poorly mixed staining solutions. To prevent this, it is essential to dissolve basic fuchsin crystals as completely as possible and to agitate samples within the staining solution periodically during staining.

If this patchiness is not present at the creation of the slide, but develops over time, it indicates too much light exposure. Exposure to concentrated fluorescent lighting for too long can cause photo bleaching in stained sections. Essentially, the basic fuchsin is burned out of the section, making it useless for microdamage analysis. It is extremely important to keep stained slides stored in the dark and to limit their light exposure during analysis.

### How important is section thickness for analysis?

First and foremost, when dealing with histological methods we must remember that we are using 2D methods to assess three‐dimensional (3D) structures. Though dubbed linear microcracks, these defects are 3D pockets within the greater microarchitecture of the bone.^60^ In 2D, we see only their length in a single plane. Furthermore, clarity under magnification is largely determined by section thickness; the thinner the section, the better light can pass through it on the microscope, the clearer the image. Most researchers use sections between 30 and 150 µm thick for microcracks studies, so it is best to use sections within that range for comparability. However, further work on the importance of section thickness and serial sections is needed.

### When studying linear microcracks, should cortical porosity be measured?

As mentioned in the discussion of histomorphometric variables, when examining linear microcracks in the context of the human rib, cortical porosity should be accounted for. However, further exploration of this question is necessary, particularly in the context of bones besides the ribs, which are less abjectly influenced by differences in metabolism and thus less prone to elevated intracortical bone loss.

### What other techniques can be used to explore microdamage?

Many methods other than light microscopy can be used to explore similar questions to those detailed here. It is established that examining diffuse damage requires more high‐powered microscopy, such as scanning electron microscopy (SEM). SEM allows the submicron measurements essential to confirm the presence of diffuse damage, which cannot be conclusively identified with light microscopy. It will be interesting to see if greater microscopic resolution can improve the reliability of interobserver and intraobserver differences.

Other imaging modalities, particularly those that rely on nondestructive 3D scanning of skeletal material, will be essential for understanding the role of linear microcracks in skeletal integrity and in living populations. Currently, synchrotron scanning, which is limited to very small samples, is the only variant of this technology with sufficient resolution to examine such features. However, as the resolution of micro–computed tomography (µCT) and clinical CT systems increases, these methods will be essential in translating bone microarchitectural research from the laboratory to the clinical setting.

## Summary

Bone's dynamic nature makes defining the importance of bone quality components a challenge and the role of microcracks demands further attention, but objective, repeatable methods are essential to move microdamage research forward. Basic fuchsin staining currently dominates linear microcrack analysis, but although there exists a variety of methods for sample preparation and data collection, available guidelines are few and often sparse in detail. With this in mind, this work provides a review of existing methods for linear microcrack analysis and provides a step‐by‐step guide for sample preparation and recommendations for data collection useful to anyone seeking to enter microcrack research. As described here, reliable staining methods are necessary to accurately capture and quantify bone microdamage, and the approach presented here has been refined over the course of years and successfully used on hundreds of rib samples. Establishing objective criteria for microcrack analysis with greater repeatability within and between observers improves research, and ultimately contributes to superior clinical approaches. Presented here are new, partially validated criteria to reduce subjectivity in linear microcrack analysis and increase comparability across studies. In addition, a photographic atlas can be found in the Supporting Information, Part II, to illustrate these criteria and to serve as a reference for further research.

The foundations of microdamage research are well laid and have been pivotal in demonstrating that microdamage forms and accumulates in bones due to loading and that accrued damage negatively affects bone strength. However, many questions remain, particularly, what role, if any, do linear microcracks play in skeletal fragility? Future work should explore “normal” in vivo damage across ages and sexes and consider the contribution of preexisting damage to bone behavior and material properties in injurious circumstances. Though current knowledge suggests that microdamage may contribute to the variance in bone material properties that leads to skeletal fragility, there is little direct evidence for this at present. Detailed analyses examining microcrack accrual relative to tested material properties, as well as cross‐sectional analysis of the spatial distribution of microdamage and other microarchitectural variables (ie, porosity, remodeling) may elucidate the loading and repair history of bone. Ultimately, understanding how microdamage acts in the context of skeletal longevity and fragility has significant clinical implications that may potentially impact how we diagnose and treat conditions in those of compromised bone health, such as those with osteopenia and osteoporosis, among others. Exploration of these and other questions requires reliable and replicable methods of sample preparation and data collection to fully capture extant microdamage. We hope the pragmatic approach presented here can work as a step toward increased method standardization and will be welcomed by bone biologists and biomechanists interested in expanding the field of bone microdamage.

## Disclosures

The authors have no potential conflicts of interest to disclose.

## Supporting information

Additional Supporting Information may be found in the online version of this article.

## References

[1] Office of the Surgeon General (US). Bone health and osteoporosis: a report of the Surgeon General. Rockville (MD): Office of the Surgeon General (US); 2004 [cited 2019 May 20]. Available from: https://www.ncbi.nlm.nih.gov/books/NBK45513/ 20945569

[2] Bolotin HH . DXA in vivo BMD methodology: an erroneous and misleading research and clinical gauge of bone mineral status, bone fragility, and bone remodelling. Bone. 2007;41(1):138–54. 17481978 10.1016/j.bone.2007.02.022

[3] Heaney RP . Is the paradigm shifting? Bone. 2003;33(4):457–65. 14555248 10.1016/s8756-3282(03)00236-9

[4] Malgo F , Appelman‐Dijkstra NM , Termaat MF , et al. High prevalence of secondary factors for bone fragility in patients with a recent fracture independently of BMD. Arch Osteoporos. 2016;11(1):12. 26906974 10.1007/s11657-016-0258-3 PMC4764633

[5] Hui SL , Slemenda CW , Johnston CC Jr . Age and bone mass as predictors of fracture in a prospective study. J Clin Invest. 1988;81(6):1804–9. 3384952 10.1172/JCI113523 PMC442628

[6] Pazianas M , van der Geest S , Miller P . Bisphosphonates and bone quality. BoneKEy Rep. 2014;3:529. 24876930 10.1038/bonekey.2014.24 PMC4037878

[7] Mashiba T , Hirano T , Turner CH , Forwood MR , Johnston CC , Burr DB . Suppressed bone turnover by bisphosphonates increases microdamage accumulation and reduces some biomechanical properties in dog rib. J Bone Miner Res. 2000;15(4):613–20. 10780852 10.1359/jbmr.2000.15.4.613

[8] Unnanuntana A , Gladnick BP , Donnelly E , Lane JM . The assessment of fracture risk. J Bone Joint Surg Am. 2010;92(3):743–53. 20194335 10.2106/JBJS.I.00919 PMC2827823

[9] Licata A . Bone density vs bone quality: what's a clinician to do? Cleve Clin J Med. 2009;76(6):331–6. 19487553 10.3949/ccjm.76a.08041

[10] Seeman E , Delmas PD . Bone quality—the material and structural basis of bone strength and fragility. N Engl J Med. 2006;354(21):2250–61. 16723616 10.1056/NEJMra053077

[11] Martin RB , Burr DB , Sharkey NA , Fyhrie DP . Skeletal tissue mechanics. 2nd ed New York: Springer, 2015.

[12] Frost HM . Presence of microscopic cracks in vivo in bone. Henry Ford Hosp Med Bull. 1960;8: 25–35.

[13] Burr DB . Targeted and nontargeted remodeling. Bone. 2002;30(1):2–4. 11792556 10.1016/s8756-3282(01)00619-6

[14] Martin RB . Is all cortical bone remodeling initiated by microdamage? Bone. 2002;30(1):8–13. 11792558 10.1016/s8756-3282(01)00620-2

[15] Allen M , Burr D . Skeletal microdamage: less about biomechanics and more about remodeling. Clin Rev Bone Miner Metab. 2008;6(1–2):24–30.

[16] Fonseca H , Moreira‐Goncalves D , Coriolano HJ , Duarte JA . Bone quality: the determinants of bone strength and fragility. Sports Med. 2014;44(1):37–53. 24092631 10.1007/s40279-013-0100-7

[17] Zimmermann EA , Ritchie RO . Bone as a structural material. Adv Healthc Mater. 2015;4(9):1287–304. 25865873 10.1002/adhm.201500070

[18] Ruff C , Holt B , Trinkaus E . Who's afraid of the big bad Wolff?: “Wolff's law” and bone functional adaptation. Am J Phys Anthropol. 2006;129(4):484–98. 16425178 10.1002/ajpa.20371

[19] Currey JD . The many adaptations of bone. J Biomech. 2003;36(10):1487–95. 14499297 10.1016/s0021-9290(03)00124-6

[20] Ritchie RO . The conflicts between strength and toughness. Nat Mater. 2011;10(11):817–22. 22020005 10.1038/nmat3115

[21] Martin RB . Fatigue damage, remodeling, and the minimization of skeletal weight. J Theor Biol. 2003;220(2):271–6. 12602399 10.1006/jtbi.2003.3148

[22] Ritchie RO , Buehler MJ , Hansma P . Plasticity and toughness in bone. Phys Today. 2009;62(6):41–7.

[23] Burr DB . The complex relationship between bone remodeling and the physical and material properties of bone. Osteoporos Int. 2015;26(3):845–7. 25526711 10.1007/s00198-014-2970-4

[24] Diab T , Condon KW , Burr DB , Vashishth D . Age‐related change in the damage morphology of human cortical bone and its role in bone fragility. Bone. 2006;38(3):427–31. 16260195 10.1016/j.bone.2005.09.002

[25] O’Brien FJ , Brennan O , Kennedy OD , Lee TC . Microcracks in cortical bone: how do they affect bone biology? Curr Osteoporos Rep. 2005;3(2):39–45. 16036100 10.1007/s11914-005-0002-1

[26] Burr DB . Why bones bend but don’t break. J Musculoskelet Neuronal Interact. 2011;11(4):270–85. 22130136

[27] Carter DR , Hayes WC . Compact bone fatigue damage: a microscopic examination. Clin Orthop Relat Res. 1977;127:265–74. 912990

[28] Diab T , Vashishth D . Morphology, localization and accumulation of in vivo microdamage in human cortical bone. Bone. 2007;40(3):612–8. 17097933 10.1016/j.bone.2006.09.027 PMC2013741

[29] Donaldson F , Ruffoni D , Schneider P , et al. Modeling microdamage behavior of cortical bone. Biomech Model Mechanobiol. 2014;13(6):1227–42. 24622917 10.1007/s10237-014-0568-6

[30] Frank JD , Ryan M , Kalscheur VL , Ruaux‐Mason CP , Hozak RR , Muir P . Aging and accumulation of microdamage in canine bone. Bone. 2002;30(1):201–6. 11792586 10.1016/s8756-3282(01)00623-8

[31] Zioupos P . Ageing human bone: factors affecting its biomechanical properties and the role of collagen. J Biomater Appl. 2001;15(3):187–229. 11261600 10.1106/5JUJ-TFJ3-JVVA-3RJ0

[32] Taylor D , Hazenberg JG , Lee TC . Living with cracks: damage and repair in human bone. Nat Mater. 2007;6(4):263–8. 17401419 10.1038/nmat1866

[33] Taylor D . Bone maintenance and remodeling: a control system based on fatigue damage. J Orthop Res. 1997;15(4):601–6. 9379271 10.1002/jor.1100150417

[34] Hazenberg JG , Freeley M , Foran E , Lee TC , Taylor D . Microdamage: a cell transducing mechanism based on ruptured osteocyte processes. J Biomech. 2006;39(11):2096–103. 16112124 10.1016/j.jbiomech.2005.06.006

[35] Herman BC , Cardoso L , Majeska RJ , Jepsen KJ , Schaffler MB . Activation of bone remodeling after fatigue: differential response to linear microcracks and diffuse damage. Bone. 2010;47(4):766–72. 20633708 10.1016/j.bone.2010.07.006 PMC2939191

[36] Verborgt O , Gibson GJ , Schaffler MB . Loss of osteocyte integrity in association with microdamage and bone remodeling after fatigue in vivo. J Bone Miner Res. 2000;15(1):60–7. 10646115 10.1359/jbmr.2000.15.1.60

[37] Lassus J , Tulikoura I , Konttinen YT , Salo J , Santavirta S . Bone stress injuries of the lower extremity: a review. Acta Orthop Scand. 2002;73(3):359–68. 12143987 10.1080/000164702320155392

[38] Frost HH . Lamellar osteoid mineralized per day in man. Henry Ford Hosp Med Bull. 1960;8: 267–72. 13825399

[39] Vashishth D , Tanner KE , Bonfield W . Experimental validation of a microcracking‐based toughening mechanism for cortical bone. J Biomech. 2003;36(1):121–4. 12485646 10.1016/s0021-9290(02)00319-6

[40] Poundarik AA , Vashishth D . Multiscale imaging of bone microdamage. Connect Tissue Res. 2015;56(2):87–98. 25664772 10.3109/03008207.2015.1008133 PMC4554347

[41] Burr DB , Hooser M . Alterations to the en bloc basic fuchsin staining protocol for the demonstration of microdamage produced in vivo. Bone. 1995;17(4):431–3. 8573418 10.1016/s8756-3282(95)00241-3

[42] Burr DB , Stafford T . Validity of the bulk‐staining technique to separate artifactual from in vivo bone microdamage. Clin Orthop Relat Res. 1990, (260):305–8. 1699696

[43] Sterchi DL . Bone In: Bancroft JD, Layton C, Suvarna SK editors. Bancroft's theory and practice of histological techniques. Oxford: Churchill Livingstone Elsevier; 2013 p. 317–52.

[44] Agnew AM , Dominguez VM , Sciulli PW , Stout SD . Variability of in vivo linear microcrack accumulation in the cortex of elderly human ribs. Bone Rep. 2017;6:60–3. 28377983 10.1016/j.bonr.2017.02.004 PMC5365308

[45] Lee TC , Myers ER , Hayes WC . Fluorescence‐aided detection of microdamage in compact bone. J Anat. 1998;193(2):179–84. 9827633 10.1046/j.1469-7580.1998.19320179.x PMC1467837

[46] Norman TL , Wang Z . Microdamage of human cortical bone: incidence and morphology in long bones. Bone. 1997;20(4):375–9. 9108359 10.1016/s8756-3282(97)00004-5

[47] Qiu S , Rao DS , Fyhrie DP , Palnitkar S , Parfitt AM . The morphological association between microcracks and osteocyte lacunae in human cortical bone. Bone. 2005;37(1):10–5. 15878702 10.1016/j.bone.2005.01.023

[48] Turnbull TL , Baumann AP , Roeder RK . Fatigue microcracks that initiate fracture are located near elevated intracortical porosity but not elevated mineralization. J Biomech. 2014;47(12):3135–42. 25065731 10.1016/j.jbiomech.2014.06.022

[49] O’Brien FJ , Hardiman DA , Hazenberg JG , et al. The behaviour of microcracks in compact bone. Eur J Morphol. 2005;42(1‐2):71–9. 16123026 10.1080/09243860500096131

[50] Burr DB , Turner CH , Naick P , et al. Does microdamage accumulation affect the mechanical properties of bone? J Biomech. 1998;31(4):337–45. 9672087 10.1016/s0021-9290(98)00016-5

[51] Danova NA , Colopy SA , Radtke CL , et al. Degradation of bone structural properties by accumulation and coalescence of microcracks. Bone. 2003;33(2):197–205. 14499353 10.1016/s8756-3282(03)00155-8

[52] Norman TL , Little TM , Yeni YN . Age‐related changes in porosity and mineralization and in‐service damage accumulation. J Biomech. 2008;41(13):2868–73. 18703196 10.1016/j.jbiomech.2008.06.032

[53] Sobelman OS , Gibeling JC , Stover SM , et al. Do microcracks decrease or increase fatigue resistance in cortical bone? J Biomech. 2004;37(9):1295–303. 15275836 10.1016/j.jbiomech.2003.12.034

[54] Agnew AM , Stout SD . Brief communication: reevaluating osteoporosis in human ribs: the role of intracortical porosity. Am J Phys Anthropol. 2012;148(3):462–6. 22419443 10.1002/ajpa.22048

[55] Agnew AM . Histomorphometry of the elderly rib: a methodological approach with implications for biomechanics, function and fracture risk Columbus, OH: Department of Anthropology, The Ohio State University; 2011 [cited 2019 May 20]. Available from: http://rave.ohiolink.edu/etdc/view?acc_num=osu1305558389

[56] Lee TC , Mohsin S , Taylor D , et al. Detecting microdamage in bone. J Anat. 2003;203(2):161–72. 12924817 10.1046/j.1469-7580.2003.00211.x PMC1571153

[57] Diab T , Vashishth D . Effects of damage morphology on cortical bone fragility. Bone. 2005;37(1):96–102. 15897021 10.1016/j.bone.2005.03.014

[58] NIH. ImageJ software. Bethesda, MD: NIH; 2018 [cited 2019 May 20]. Available from: https://imagej.nih.gov/ij/

[59] Dominguez VM , Agnew AM . The use of ROI overlays and a semi‐automated method for measuring cortical area in ImageJ for histological analysis. Am J Phys Anthropol. 2019;168(2):378–82. 30597528 10.1002/ajpa.23747

[60] Larrue A , Rattner A , Peter ZA , et al. Synchrotron radiation micro‐CT at the micrometer scale for the analysis of the three‐dimensional morphology of microcracks in human trabecular bone. PLoS One. 2011;6(7):e21297. 21750707 10.1371/journal.pone.0021297 PMC3131277

[61] Schaffler MB , Choi K , Milgrom C. Aging and matrix microdamage accumulation in human compact bone. Bone. 1995;17:521–5. 8835305 10.1016/8756-3282(95)00370-3

[62] Boyce TM , Fyhrie DP , Glotkowski MC , Radin EL , Schaffler MB. Damage type and strain mode associations in human compact bone bending fatigue. J Orthop Res. 1998;16:322–9. 9671927 10.1002/jor.1100160308

[63] Huja SS , Hasan MS , Pidaparti R , Turner CH , Garetto LP , Burr DB . Development of a fluorescent light technique for evaluating microdamage in bone subjected to fatigue loading. J Biomech. 1999;32:1243–9. 10541076 10.1016/s0021-9290(99)00047-0

