# Supplementary material for: Microdamage as a Bone Quality Component: Practical Guidelines for the Two‐Dimensional Analysis of Linear Microcracks in Human Cortical Bone
Source: JBMR Plus. 2019 Jul 17;3(6):e10203. doi: 10.1002/jbm4.10203 (PMC6636773; doi:10.1002/jbm4.10203)
Supplement: Supplementary file 1 — Additional Supporting Information may be found in the online version of this article. [file JBM4-3-na-s001.docx]

**Consumables and equipment for preparation and analysis of linear microcracks**

For ease of reference, a list of the consumables and equipment used in the Skeletal Biology Research Laboratory are provided here. While other materials can be substituted, this list is provided as a starting point for anyone interested in pursuing traditional 2D linear microcrack analysis.

*Sample preparation equipment*

Staining

- JT Baker basic fuchsin
- Scale and weigh paper
- Ethanol (100% can be diluted with distilled water to various grades)
- Distilled water
- 1000 mL glass beakers
- Magnetic stir plates and stir bars
- Nylon biopsy bags
- Staples
- Pencil (preferably traditional graphite, not mechanical)
- Paper labels
- Vacuum pump

Embedding

- Methyl methacrylate (MMA)
- Dibutyl phthalate
- Perkadox
- Film canisters
- Water proof tape
- Vacuum pump with condenser

Sectioning

- Diamond wire saw
- Diamond wire
- Glass slides
- Binder clips
- Kimwipes

Mounting

- Glass slides (25x75 mm preferred for ribs)
- Glass coverslips (22x22 mm preferred for ribs)
- Eukitt mounting medium
- Glass rod
- Xylene
- Fine point forceps
- Cardboard slide drying trays

*Data collection equipment*

- Olympus BX63 microscope with bright field and fluorescent capabilities
- Olympus DP73 camera
- CellSens imaging software
- ImageJ software (NIH)
- Various objectives (4x, 10x, 20x)
- Wacom digitizing tablet
