# Supplementary material for: Microdamage as a Bone Quality Component: Practical Guidelines for the Two‐Dimensional Analysis of Linear Microcracks in Human Cortical Bone
Source: JBMR Plus. 2019 Jul 17;3(6):e10203. doi: 10.1002/jbm4.10203 (PMC6636773; doi:10.1002/jbm4.10203)
Supplement: Supplementary file 2 [file JBM4-3-na-s002.pdf]

Linear Microcrack Criteria and Photographic Atlas

Victoria M. Dominguez

September 25, 2017

Classification criteria.. 2
Figure 1 3
Figure 2 4
Figure 3 4
Figure 4 5
Figure 5 6
Figure 6 7
Figure 7 8
Figure 8 9
Figure 9 9
Figure 10 10
Figure 11 10
Figure 12 11
Figure 13 11
Figure 14 12
Figure 15 12

Classification criteria

The classification criteria laid out below are for the assessment of linear microcracks. These criteria are based in part on the definitions laid out by Burr and Stafford (1990) and revised in Lee et al. (1998), but also include additional considerations for reliable counting.

- A linear microcrack is a sharply defined line with a halo of basic fuchsin. Linear microcracks are more robust in appearance than canaliculi, which often resemble a spiderweb criss-crossing the bone, but smaller than vascular channels like Volkmen's canals, which are wider. (Figs. 1—3, 5)
- Only cracks that visibly open on the surface of the section being analyzed should be counted. When viewed under the microscope, the feature in question should be counted if it appears to open when the depth of focus is changed, if not, it should be excluded. This is best seen in bright field. (Fig. 4)
- Microcracks that are not artifactual will be stained through the depth of the section, appearing darker than the surrounding area. (Figs. 6 and 7)
- Single cracks that are distinguishable from tip to tip should be counted as one. Length measurements should follow the path of the crack (Fig. 8).
- In some cases, cracks appear to coalesce, resulting in what looks like tree branching. In these instances, the longest axis should be counted as one and measured as the primary crack. Branches should be counted as independent cracks, with each branch measured from the point of intersection with the primary crack to their free end (Fig. 9).

Additional criteria:

- Microcracks are sometimes seen to be partially stained. This is attributed to one of two causes: (1) the stain did not fully impregnate the existing microcrack during the staining process, or (2) the existing microcrack continued to propagate during the process of slide preparation after the staining process was completed, resulting in a portion of the microcrack being unstained.
  (1) If the crack is not stained through the depth of the section, do not count it. (Figs. 6, 7, 10)
  (2) If a part of the crack is stained through the depth of the section, the microcrack should be counted, but only the stained portion should be measured. (Figs. 11 and 14)
- Debonding of lamellar layers presents with a similar appearance to linear microcracks. This is generally seen between layers of concentric lamellae (within the secondary osteons) or longitudinal layers of lamellar apposition (at the periosteal borders of the bone). Debonding should not be counted as microcracks, with two exceptions. (Fig. 12)
  (1) If the plane of separation is great enough that it appears open on the surface of the section, it should be counted and measured. (Fig. 13)
  (2) If the microcracks run into and propagate through such planes of separation, it should be counted. This happens most often when microcracks break through or deflect around an osteon's reversal line. (Fig. 14)
- Instances where a series of fine lines that are difficult to distinguish are seen to run through each other are known as cross-hatching. Damage that bears a cross-hatched appearance is not open to the section surface and cannot be reliably traced, and therefore should not be counted in linear microcrack assessment. (Fig. 15)

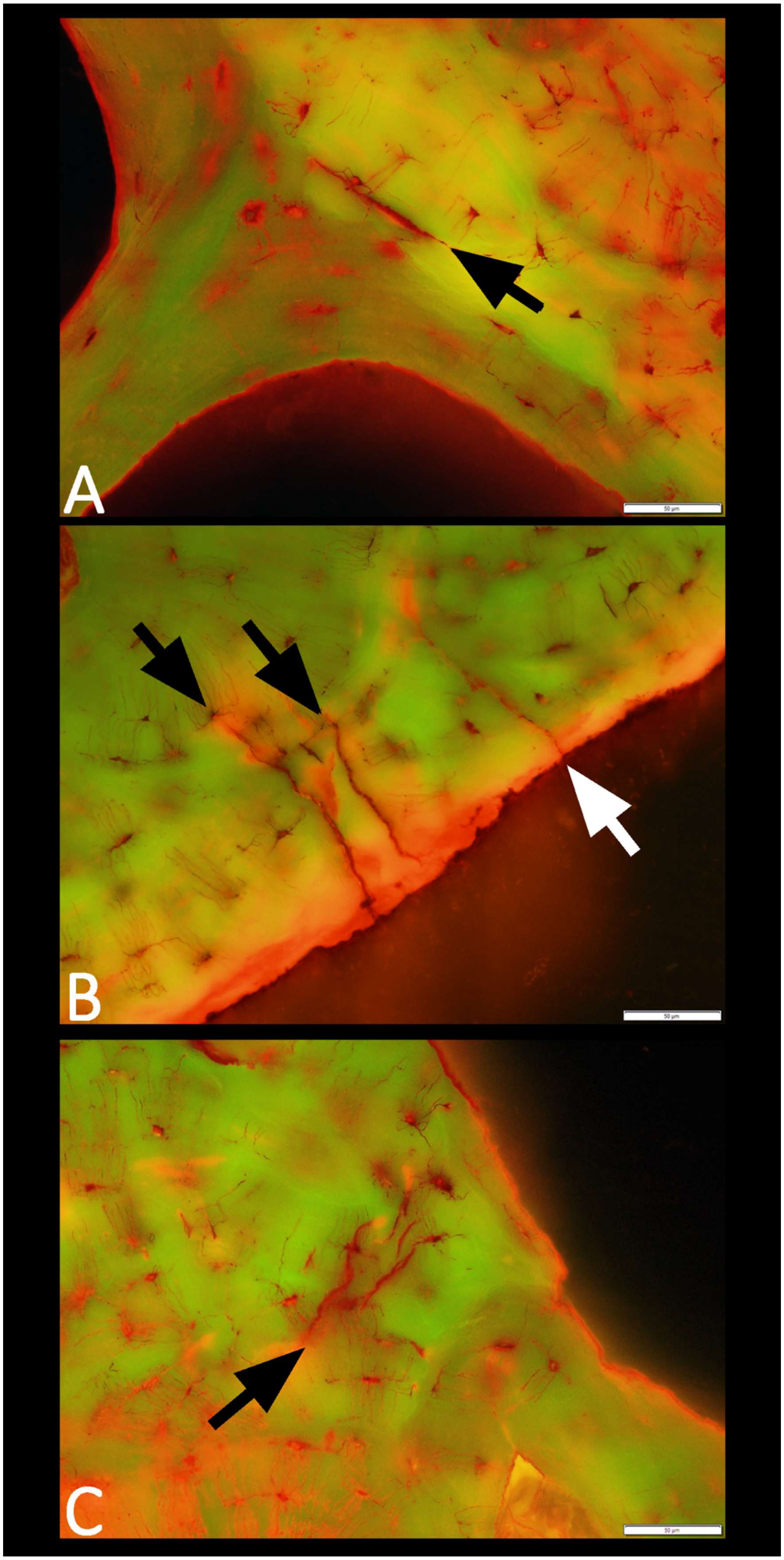

Figure 1 Linear microcracks. A) A single microcrack in the cortex near the endosteal border, indicated by the black arrow. B) Multiple linear microcracks radiating near the periosteal border. The two microcracks on the left are stained through the depth of the section (black arrows), while the microcrack on the right is incompletely stained (white arrow). C) A small cluster of microcracks that are still individually distinguishable.

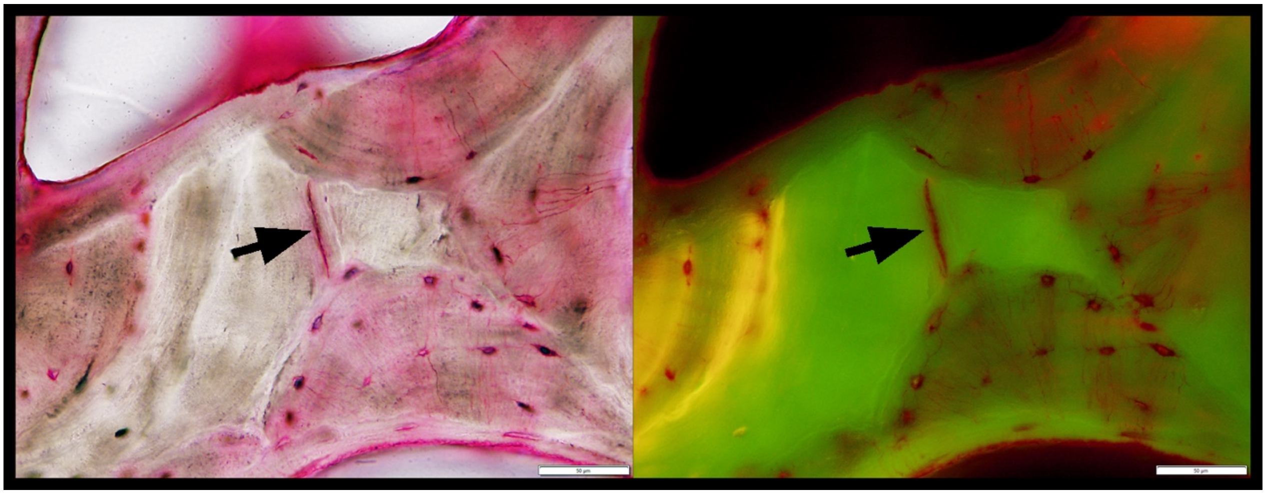

Figure 2 Linear microcrack illuminated in bright field (left) and fluorescence (right). Unstained portions appear green in FITC fluorescence, while the cracks, osteocytic lacunae, Haversian canals, and canaliculi are red due to their uptake of basic fuchsin.

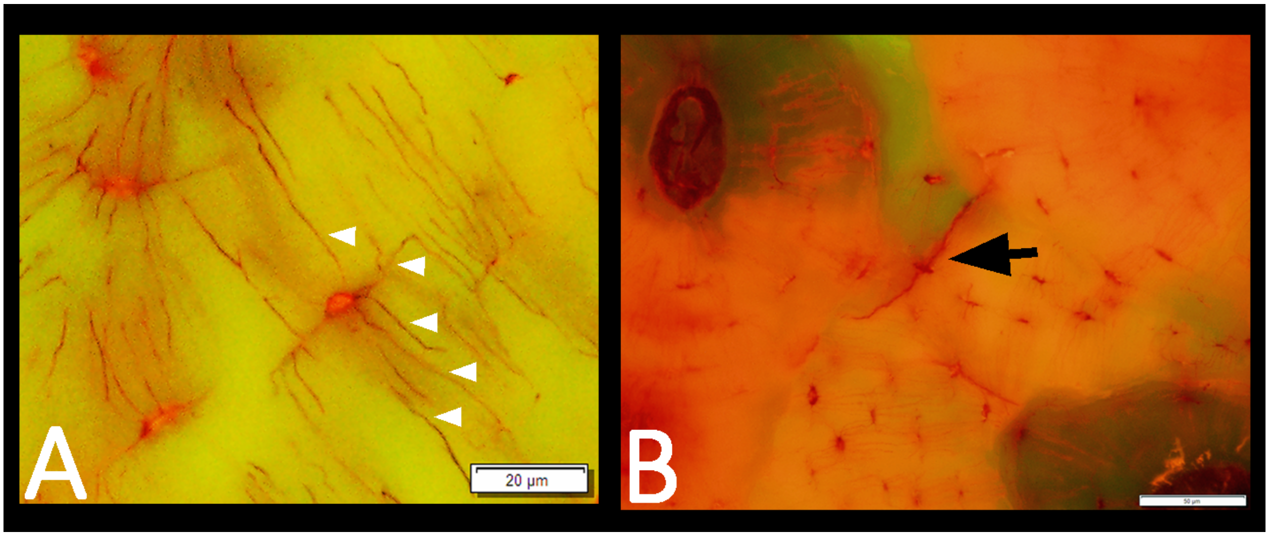

Figure 3 A) Close-up of canaliculi connecting osteocytic lacunae, which often take up stain and should not be mistaken for linear microcracks (white arrows). B) Slightly less magnified example of a linear microcrack running through an osteocytic lancuna (black arrow). It is larger, wider, and has more distinct edges than the canaliculi pictured in A.

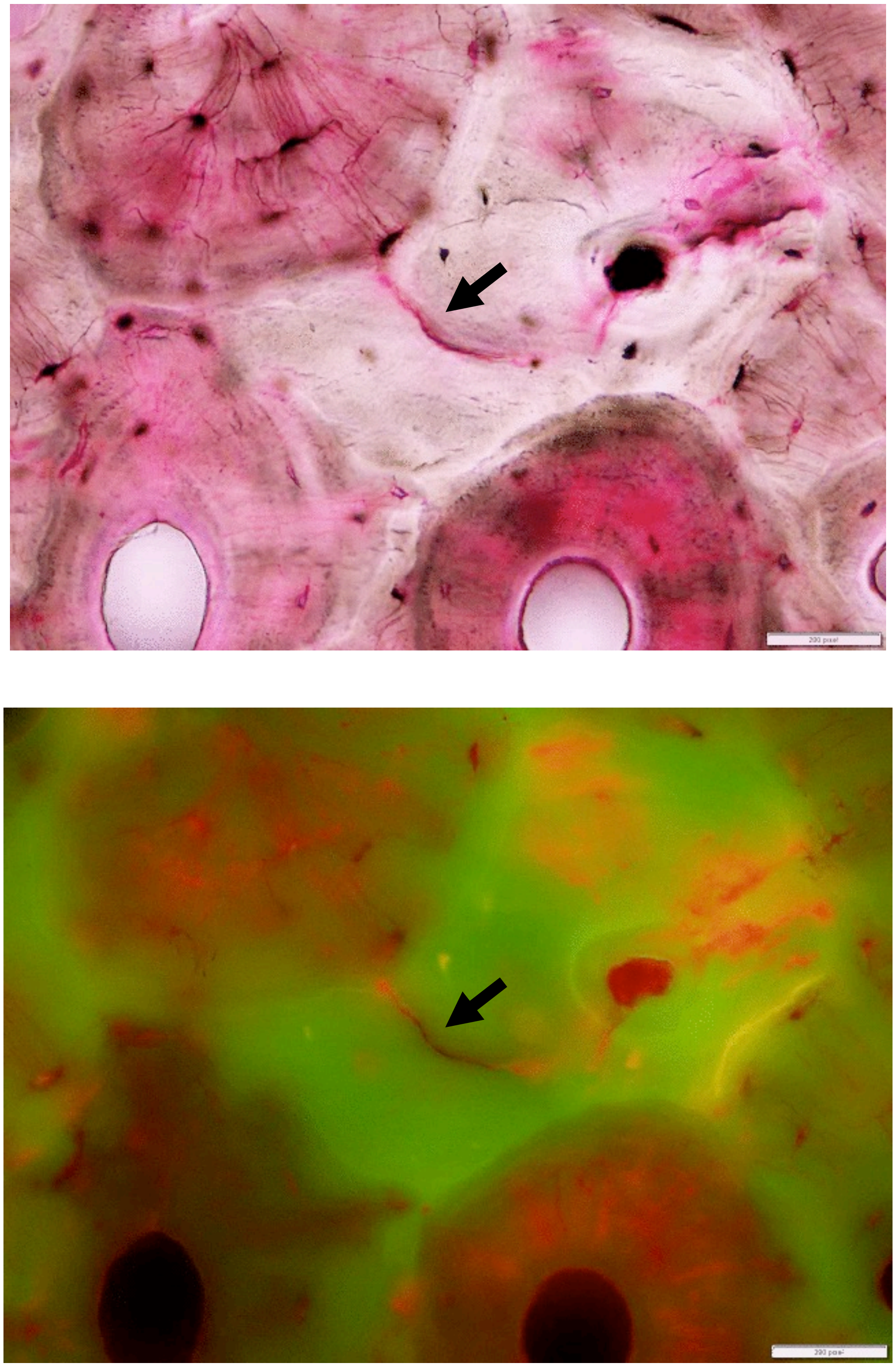

Figure 4 GIFs demonstrating what a crack open on the surface looks like in both bright field (top) and fluorescent (bottom) lighting. Double click on an image to view the animation. As the depth of focus changes, the edges of the crack appear sharper, indicating that the crack is open on the observed plane. This is best seen under bright field illumination.

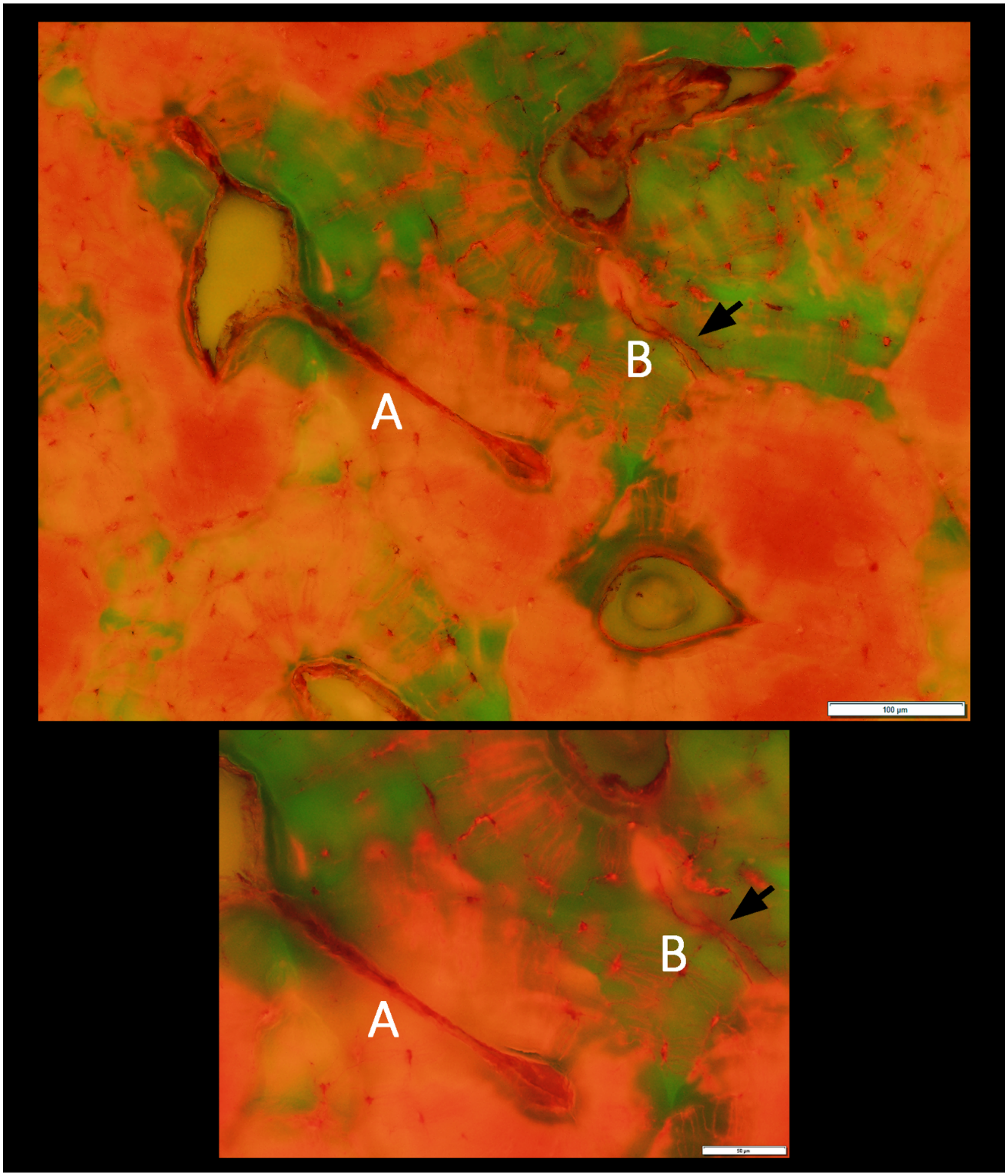

Figure 5 Comparison of a Volkman's canal (A) and two linear microcracks (B, indicated by black arrows). The Volkman's canal is wider, and when the image is zoomed out (top), it can clearly be seen to work as a transverse connection between the Haversian canals of the two secondary osteons that surround it.

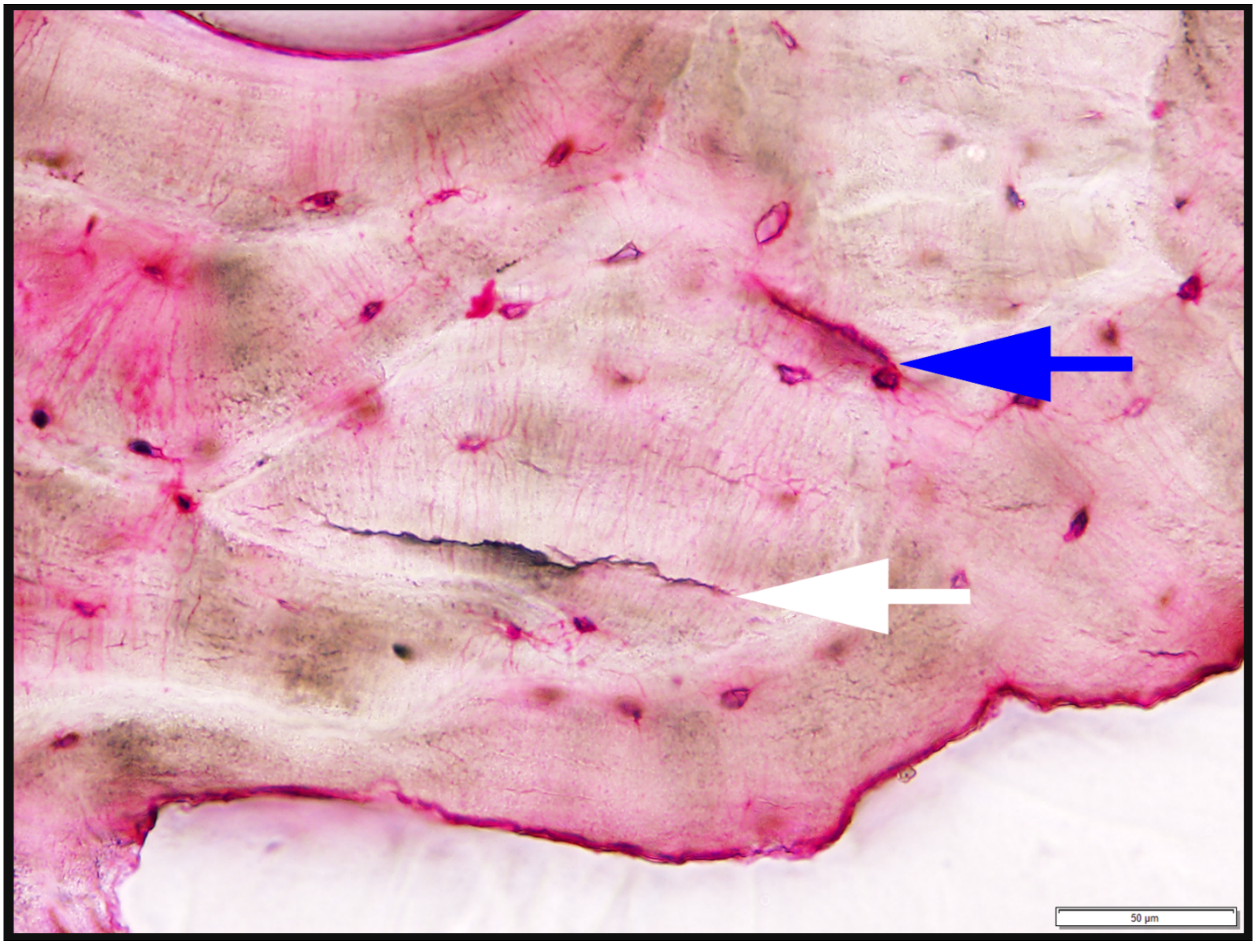

Figure 6 A bright field image showing a linear microcrack resulting from in vivo *conditions (blue arrow) versus an artefactual microcrack resulting from sample preparation (white arrow). The blue arrow indicates a linear microcrack that is stained through its depth and surrounded by a halo of basic fuchsin, while the white arrow shows a crack with sharp edges but lacking any stain through its depths or surrounding it.*

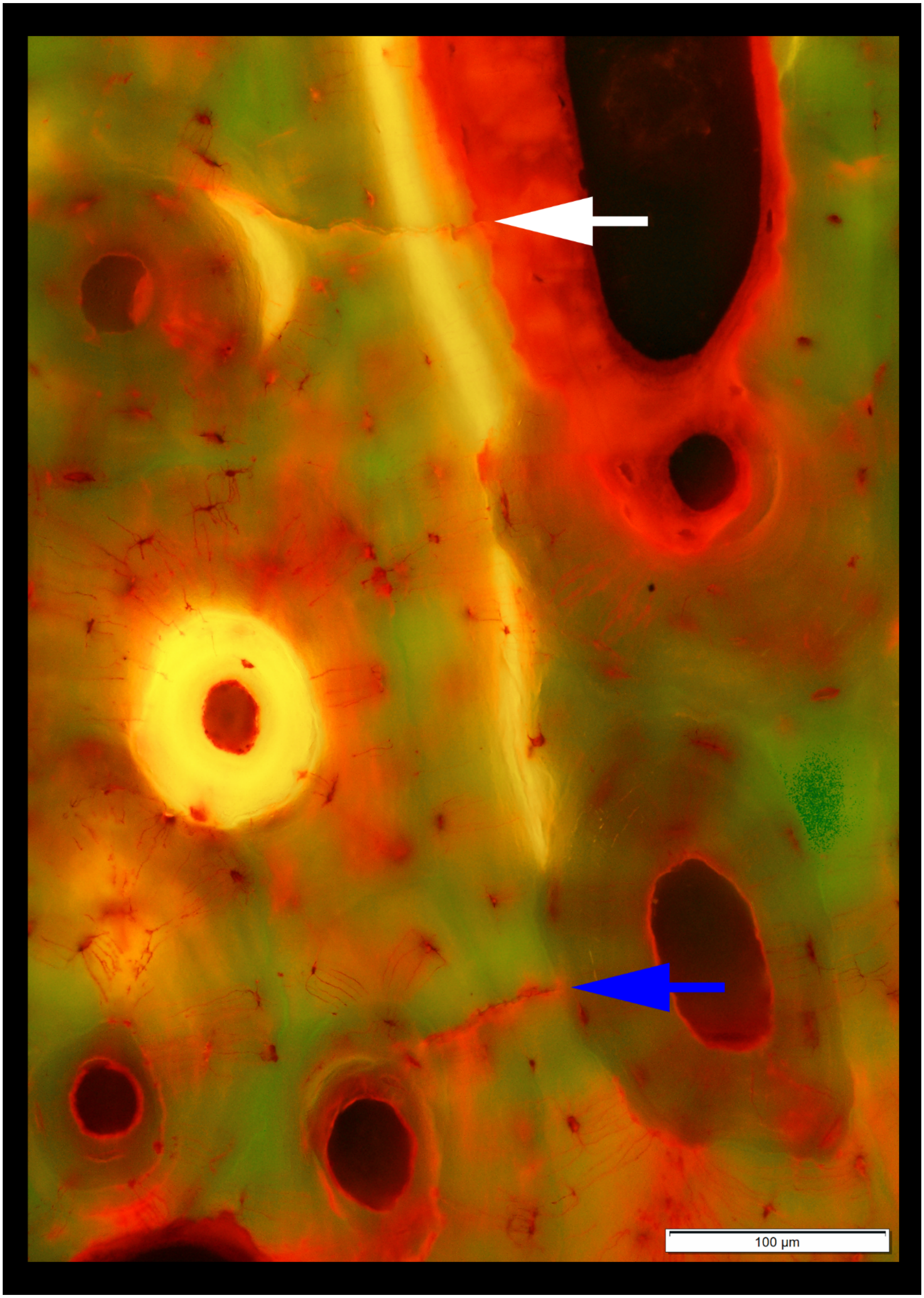

Figure 7 Unstained versus stained linear microcracks under fluorescence. The blue arrow is pointing to an in vivo *microcrack, indicated by the visible opening of the crack and the clear uptake of stain, which gives it that bright red/orange appearance under epifluorescence. The white arrow is pointing to a microcrack that likely stemmed from processing of the sample after en bloc staining was completed and should not be counted or measured.*

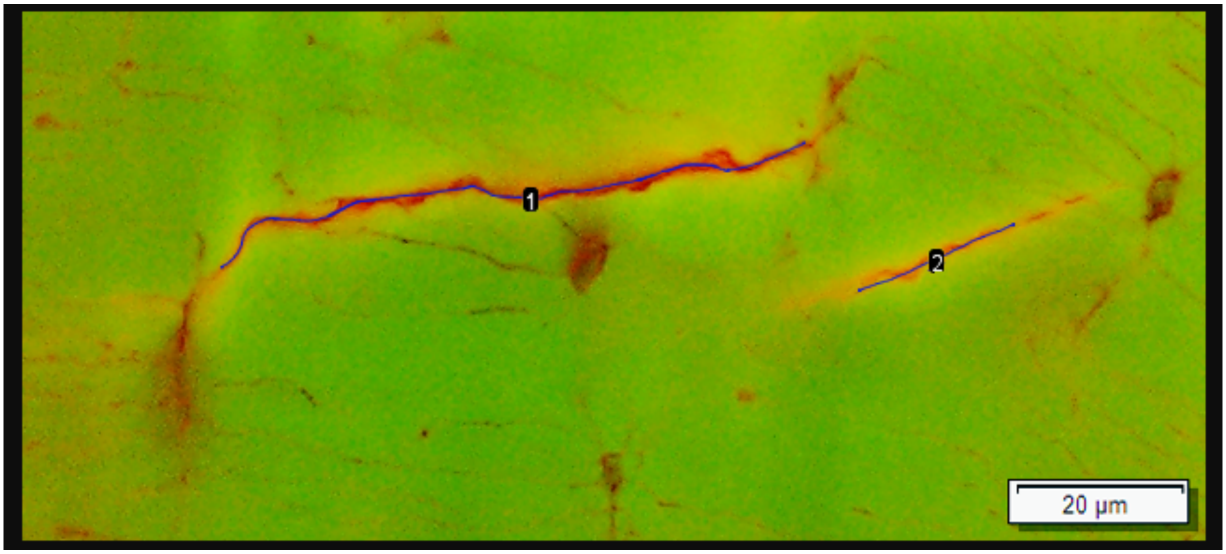

Figure 8 An example of how to measure linear microcracks. When a discrete linear microcrack is visible, the length of the crack should be traced from end to end, following the curves of the crack. Each crack here is traced with a dark blue line.

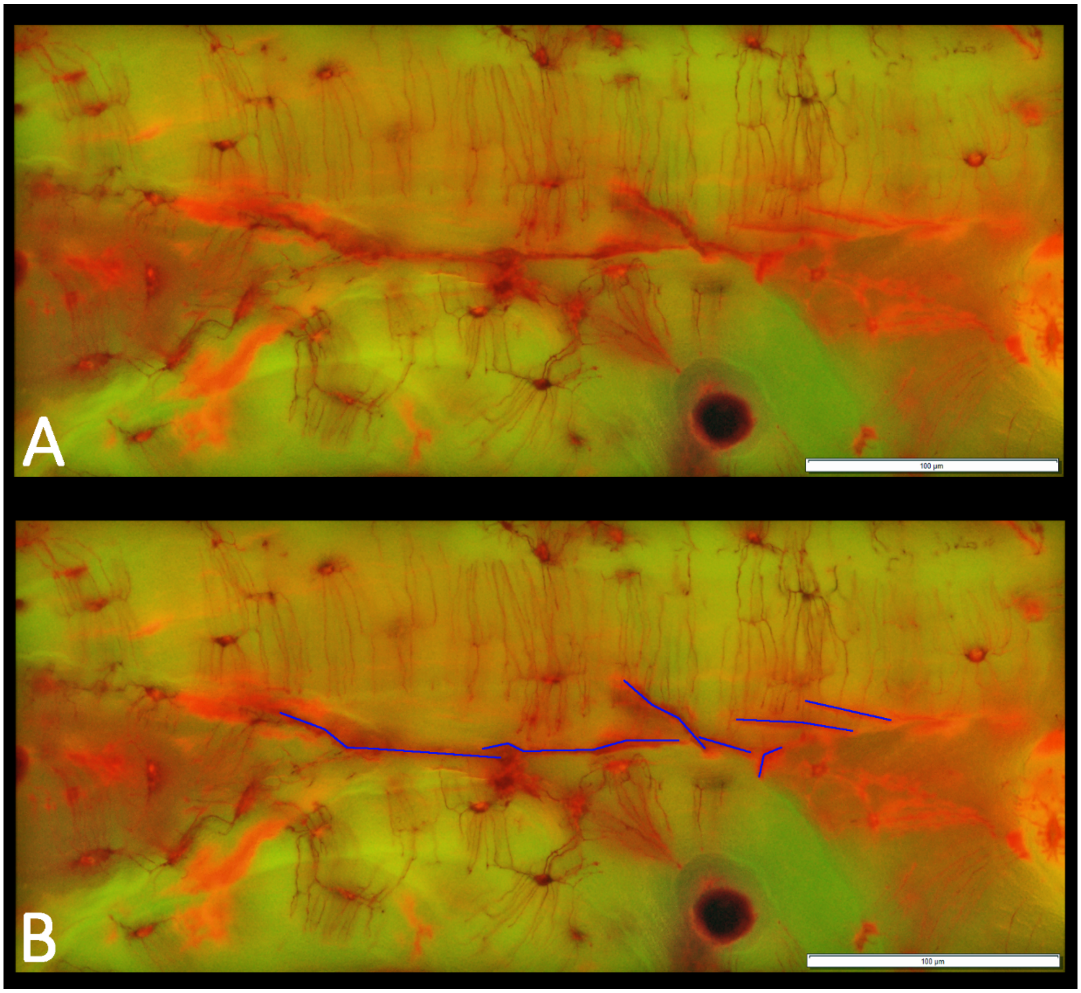

Figure 9 A) Complex clustering of linear microcracks that appear to run together and branch off each other. B) The same image as pictured in A, but now showing how measurements would be taken according to the criteria laid out here. The primary crack will be defined along its longest axis and any additional cracks will be measured as branches from this line. Each crack is marked by a distinct dark, blue line.

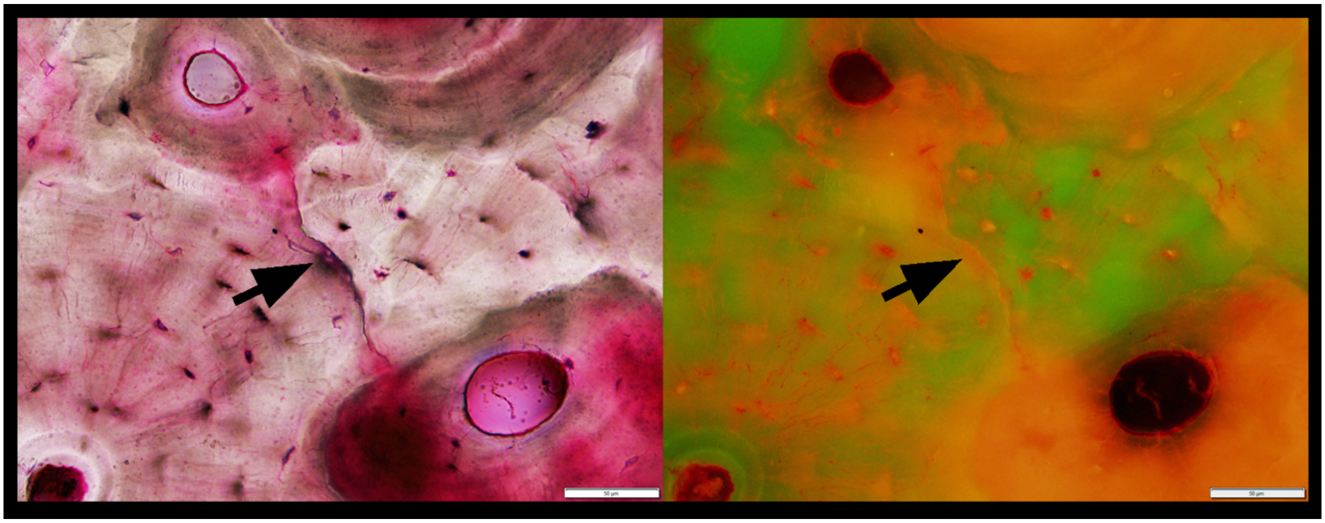

Figure 10 Bright field (left) and fluorescent (right) images of a linear microcrack that is not stained through the depth of the section. Though there is some stain uptake, it is clear that the basic fuchsin has not permeated the depth of the section and any evidence of a halo is minimal and constrained to the ends of the crack. Such a crack is deemed artifact and should not be included in linear microcrack analyses.

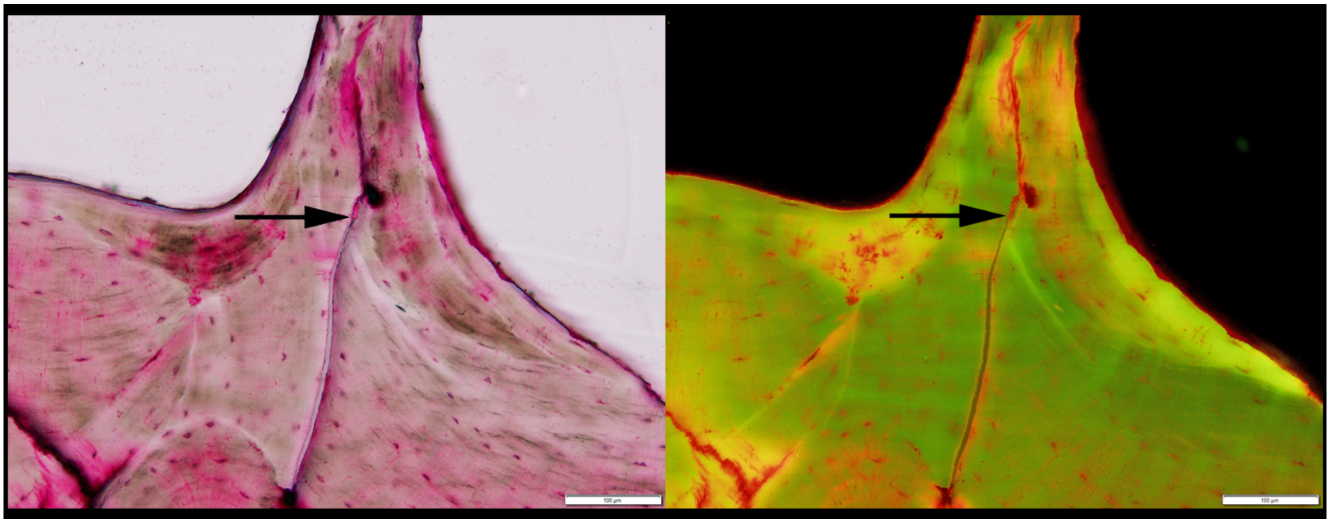

Figure 11 Bright field (left) and fluorescent (right) images of a crack that is partially stained. In this linear microcrack, the upper portion is infused with basic fuchsin, while the lower portion is not. The point at which the stain ceases is indicated by the black arrow. Above the arrow is a crack acquired in vivo*, as indicated by the presence of basic fucshin, while below the arrow is artifcatual damage. It is likely that the* in vivo *crack continued to propagate during slide preparation. As such, the stained portion of the crack should be counted and measured, while the unstained portion should be excluded from analysis.*

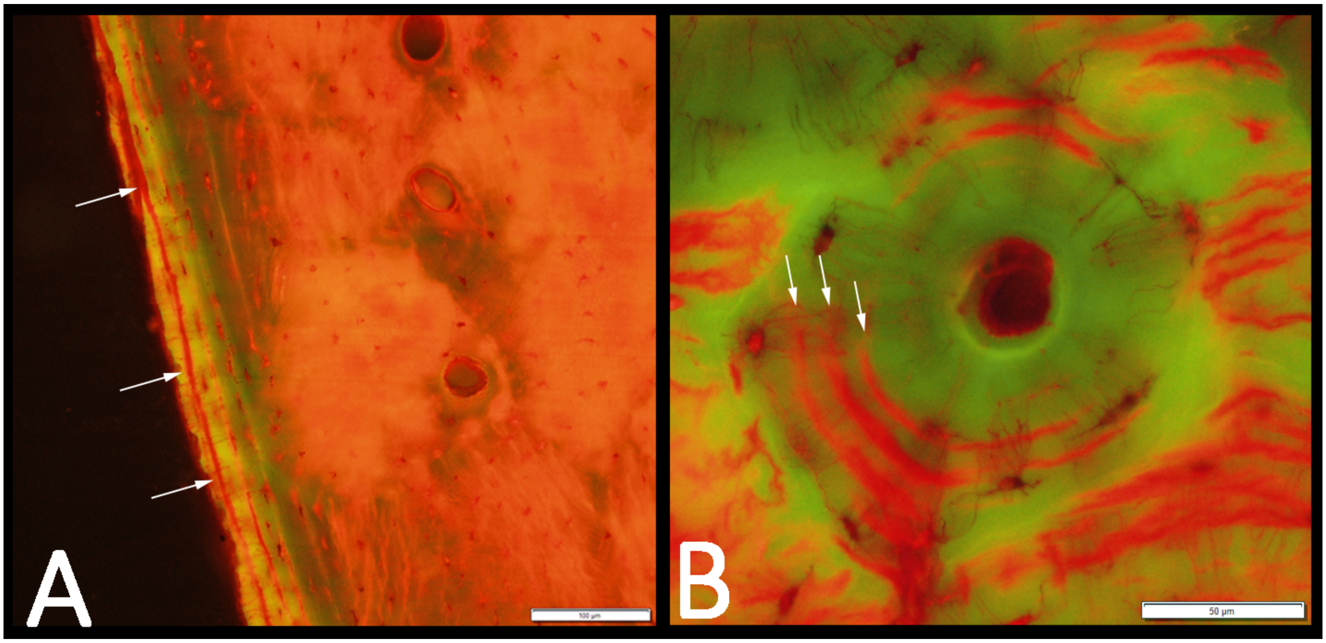

Figure 12 A) Example of debonding between the appositional layers of lamellae at the periosteal border, highlighted by the four white arrows. B) Example of debonding between the layers of circumferential lamellae within a secondary osteon, highlighted by the three white arrows. Though the stain uptake indicates that there is damage in these regions, the observed examples do no open onto the surface of the section and should not be counted.

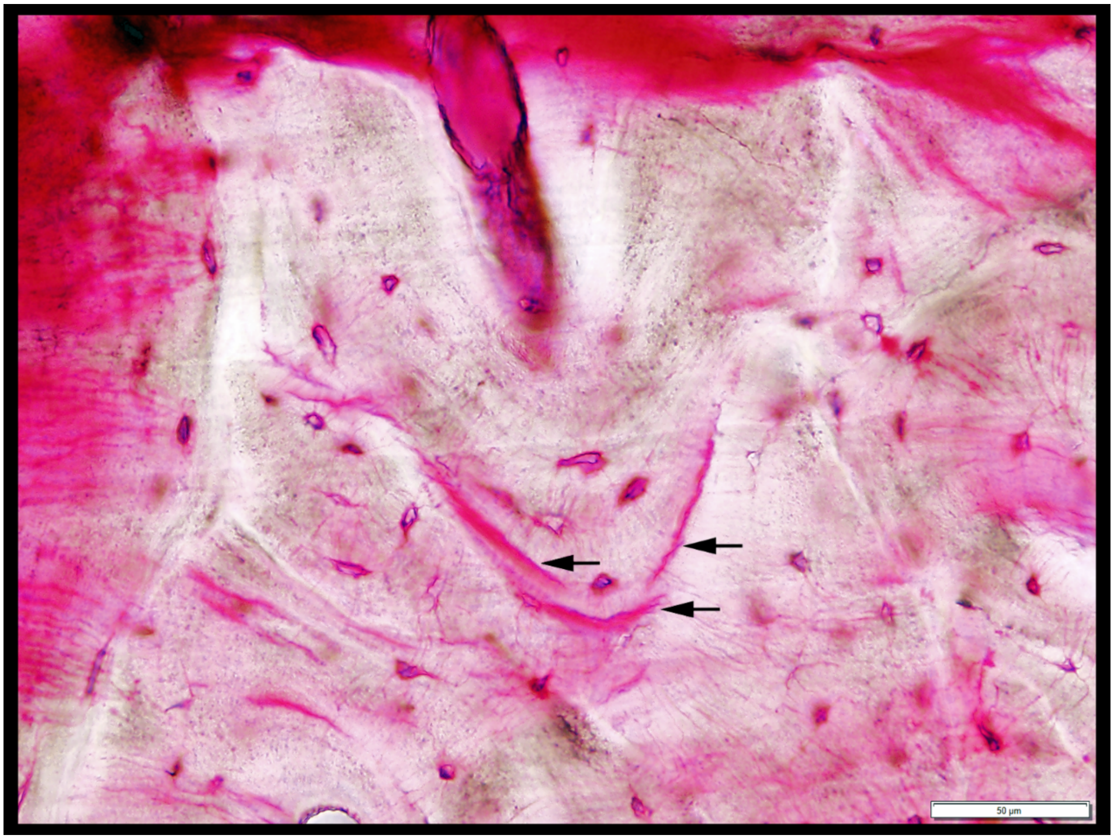

Figure 13 Similar to Figure 12B, this image demonstrates debonding between the lamellar layers of a secondary osteon. However, as seen in the bright field image, the sharp borders of an open crack are visible, meaning that in this instance, the damage should be counted during analysis. Linear structures that do not open onto the surface of a section likely reflect an open defect on another plane of the section.

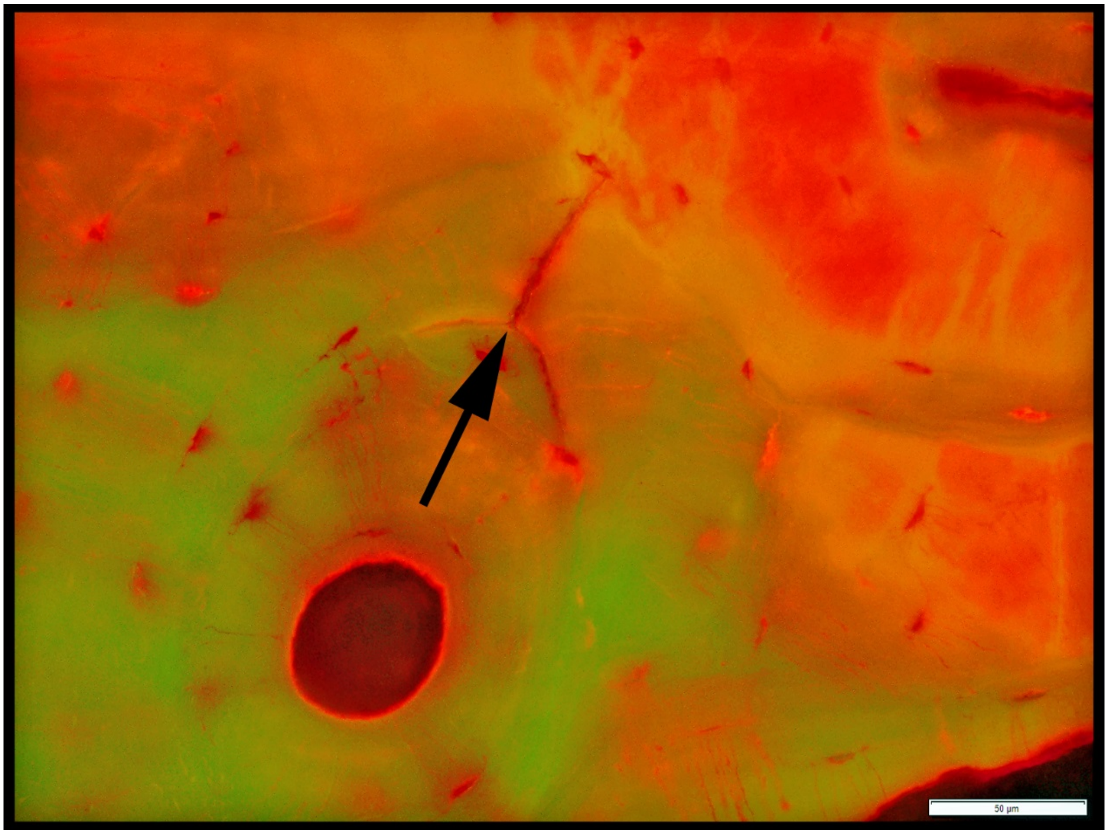

Figure 14 Example of a linear microcrack hitting the reversal line of secondary osteon and being deflected. Stain uptake indicates that the crack likely propagated to the right of the arrow in vivo, but that the opening of the crack to the left of the arrow may have resulted after the staining process during the course of sample preparation. In this case, the opening to the left should not be included in counts or measures.

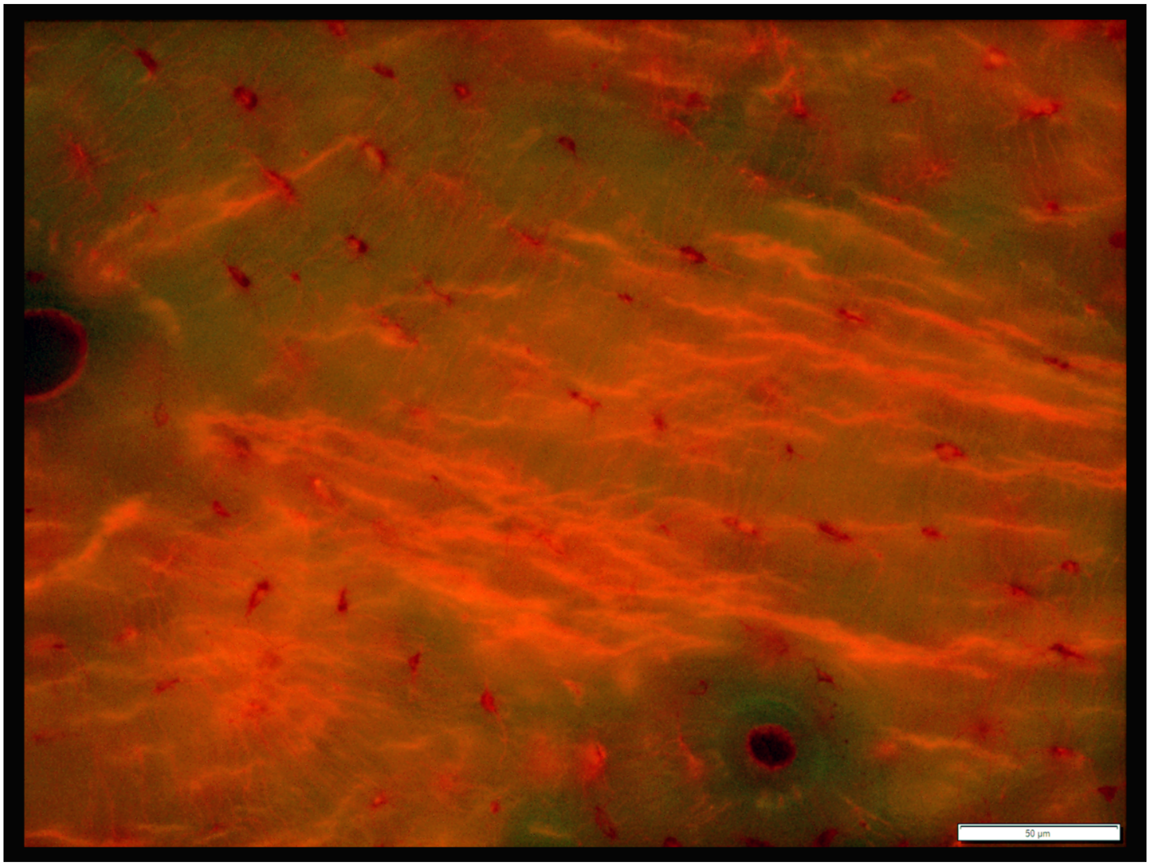

Figure 15 Example of cross-hatching, which resembles linear microcracks, but does not open onto the surface and has indistinct borders. This type of damage should not be included in counts and measures of linear microcracks.
